# Two macrocycle-based sensors for anions sensing

**DOI:** 10.1038/s41598-018-36916-w

**Published:** 2019-01-24

**Authors:** Yingjie Liu, Zhixiang Zhao, Ran Huo, Qingxiang Liu

**Affiliations:** 1 Tianjin Key Laboratory of Process Measurement and Control, Institute of Robotics and Autonomous Systems, Tianjin University, Tianjin, 300072 China; 2 Key Laboratory of Inorganic-Organic Hybrid Functional Materials Chemistry (Tianjin Normal University), Ministry of Education; Tianjin Key Laboratory of Structure and Performance for Functional Molecules, College of Chemistry, Tianjin Normal University, Tianjin, 300387 China

## Abstract

Two macrocyclic bis-benzimidazolium salts 2 and 4 (23-membered for 2 and 25-membered for 4) were prepared, and their structures were confirmed by X-ray crystallography, ^1^H NMR and ^13^C NMR spectroscopy. The research of anion recognitions using 2 or 4 as hosts were carried out with the methods of fluorescence and ultraviolet spectroscopy, ^1^H NMR titrations, MS and IR spectra. The experiment results show that 2 can detect acetate anion and 4 can detect nitrate anion with favorable selectivity and sensitivity.

## Introduction

Anion recognition has attracted extensive attention in host-guest chemistry^1–3^. Among numerous anions, acetate group and nitrate group play very important roles in medical, biological and environmental areas. For example, sodium acetate can restrain the growth of microorganism and lengthen the shelf life of food^4–11^. Also, acetate group can participate in metabolic reactions in human body^12,13^.

Nitrate salt as a pollutant exists extensively in the natural world, and it can be absorbed by crops from polluted water and soil. After these crops are consumed by human, the nitrate salt can be transformed into poisonous nitrite salt^14–18^. Hence, the detection of acetate group and nitrate group is of great importance for improving human health and protecting the environment. Some methods for detecting acetate group and nitrate group have been reported, such as fluorescence spectroscopy^19–22^, ultraviolet spectroscopy^23–25^ and ion chromatography^26,27^. By contrast, fluorescence method has been turned out to be simpler, more sensitive and easier to operate^28–30^. It is known that the change of fluorescence spectroscopy of host is mainly related to the interactions between host and guest. After the guest is added to the host, if fluorescence spectroscopy of host has remarkable change (such as enhancement, decrease or shift of emission peak), which shows that there exist strong interactions between the host and the guest. Otherwise, there are not obvious interactions between the host and the guest. The interaction forces between the host and the guest include mainly H-bonds, anion-π interactions and electrostatic interactions and so on^31–37^, in which H-bonds are common interactions. To form H-bonds between the host and the guest, the introduction of some specific binding sites in the design of host is necessary (such as urea, thiourea, amide and imidazolium or benzimidazolium groups)^38–42^.

In the process of seeking appropriate hosts, cyclic compounds with two benzimidazolium groups come into our sight. Because this type of hosts is good H-bond donors, they can combine with anionic guests through C-H···X hydrogen bonds^4,43,44^. In the paper, two new macrocyclic compounds with benzimidazolium moieties (**2** and **4**) are reported. The structures of 23-membered macrocycle **2** and 25-membered macrocycle **4** are confirmed by X-ray crystallography and ^1^H NMR, ^13^C NMR spectroscopy. The research of anion recognitions using **2** and **4** as hosts were carried out with the method of fluorescence spectroscopy, ^1^H NMR titrations, HRMS and IR spectra.

## Results and Discussion

### Preparation and structures of macrocyclic compounds 2 and 4

**Figure 1:**
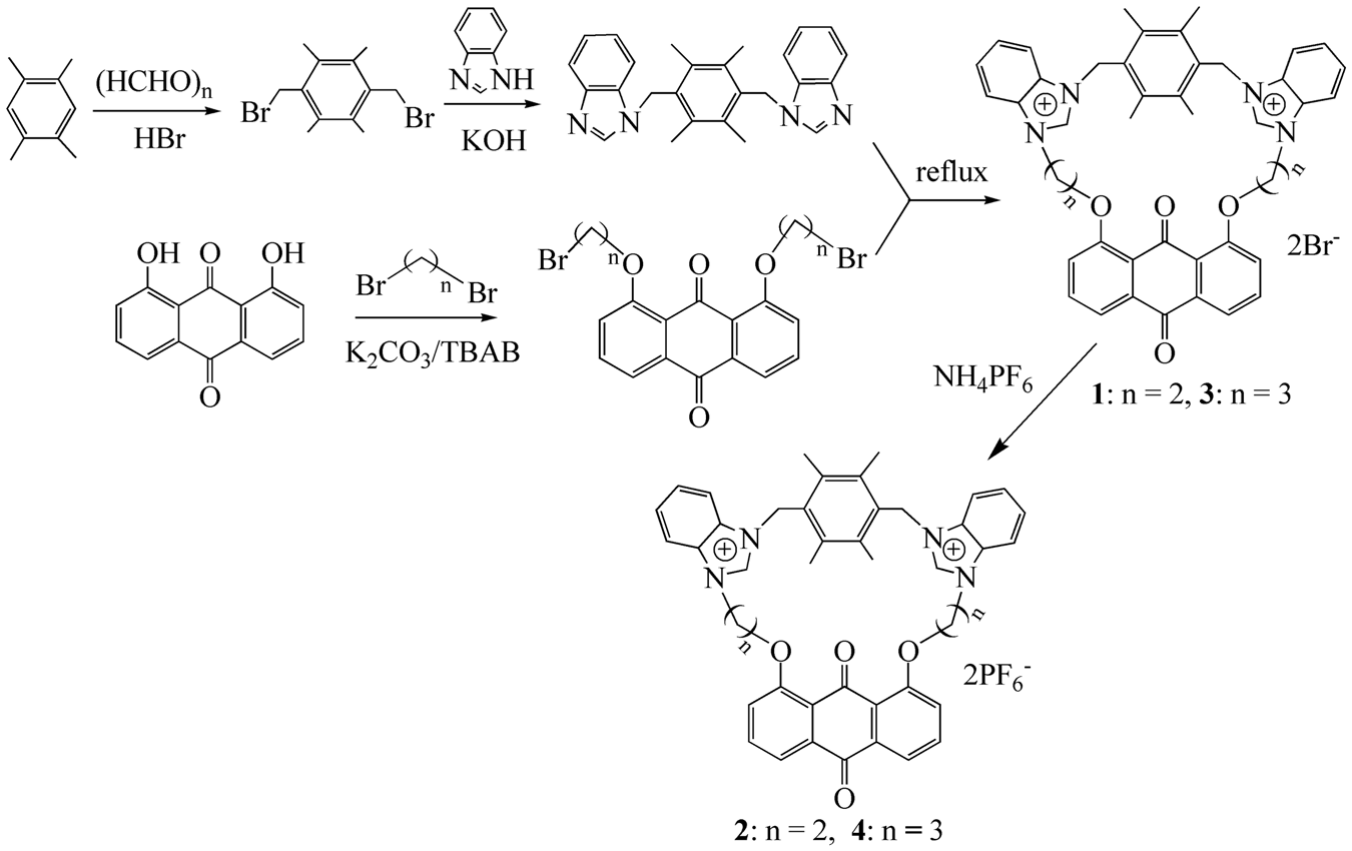
Preparation of macrocyclic compounds **2** and **4**.

As shown in Fig. 1, the durene reacted with HBr and paraformaldehyde to give 1,4-di(bromomethyl)-durene, and then the reaction of 1,4-di(bromomethyl)-durene with KOH and benzimidazole afforded 1,4-di(benzimidazol-1-yl-methyl)-durene. 1,8-di(2′-bromoethoxy)-9,10-anthraquinone or 1,8-bis(3′-bromopropoxy)-9,10-anthraquinone were gotten through the reaction of 1,8-dihydroxy-9,10-anthraquinone with 1,2-dibromoethane or 1,3-dibromopropane, and they further reacted with 1,4-di(benzimidazol-1-yl-methyl)-durene to generate macrocyclic compounds **1** and **3** with two bromide anions. Compounds **2** or **4** (n = 2 for **2** and n = 3 for **4**) were formed through the anion exchange reaction of compounds **1** or **3** with NH_4_PF_6_. Compounds **2** and **4** demonstrate excellent stability to air, moisture and heat, and their solubility are good in strong polar solvents like CH_3_CN, CH_2_Cl_2_ and DMSO, but they are almost insoluble in nonpolar organinc solvents such as benzene and hexane. In the ^1^H NMR spectra of **2** and **4**, the chemical shift of benzimidazolium proton signals (NC*H*N, *δ* = 8.82 ppm and 8.41 ppm) accords with the chemical shifts of known azolium salts^45–52^.

**Figure 2:**
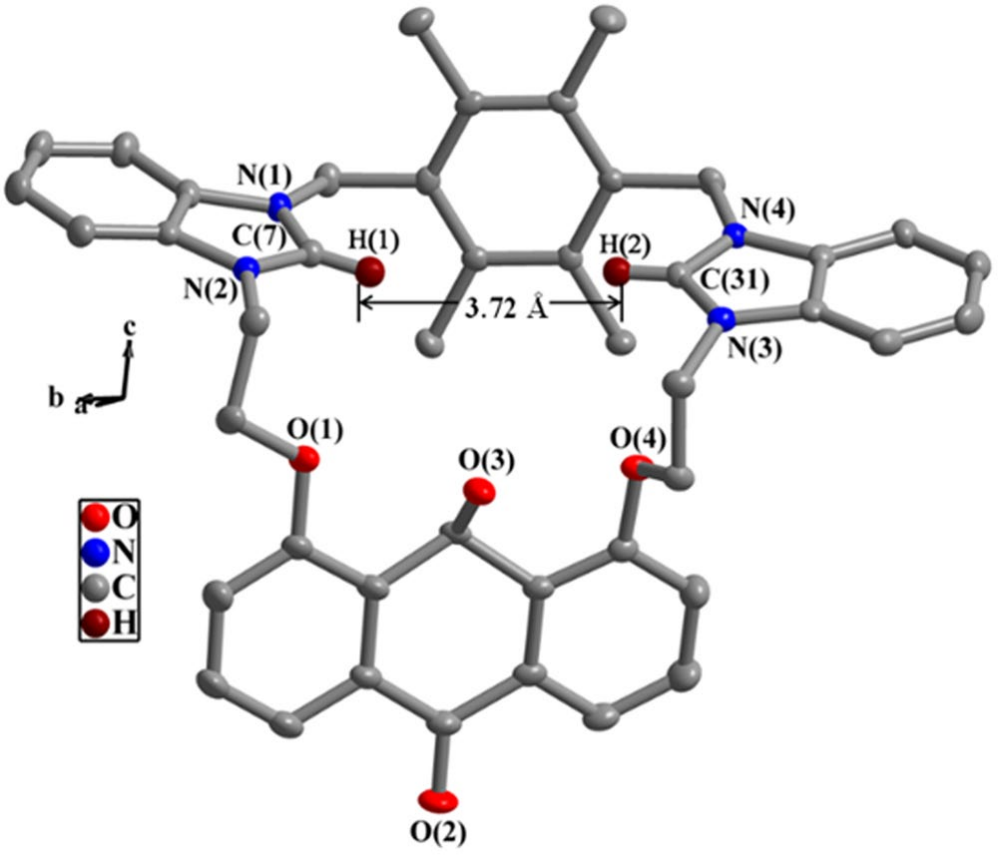
Crystal structure of compound **2**. Some bond lengths (Å) and angles (°): N(1)-C(7) 1.336(2), N(2)-C(7) 1.328(8), N(3)-C(31) 1.329(5), N(4)-C(31) 1.326(8); N(1)-C(7)-N(2) 110.3(2), N(3)-C(31)-N(4) 111.0(2).

**Figure 3:**
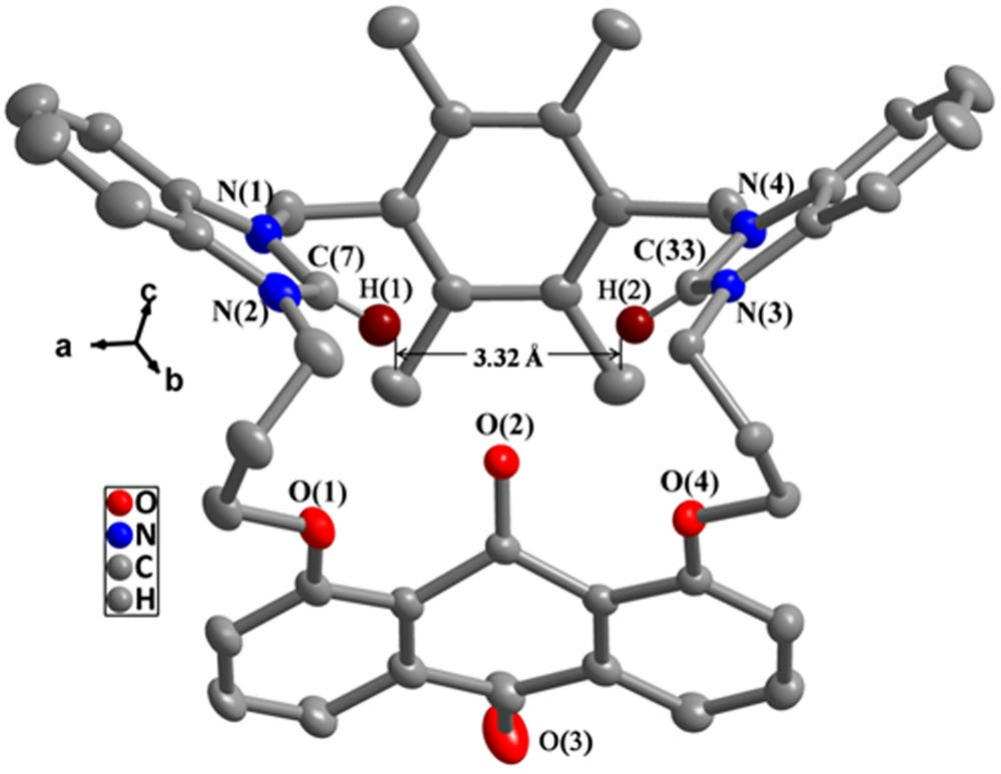
Crystal structure of compound **4**. All hydrogen atoms were omitted for clarity. Selected bond lengths (Å) and angles(°): N(1)-C(7) 1.330(5), N(2)-C(7) 1.324(5), N(3)-C(33) 1.320(4), N(4)-C(33) 1.323(4); N(1)-C(7)-N(2) 111.1(3), N(3)-C(33)-N(4) 111.2 (3).

23-Membered and 25-membered macrocycles in the molecular structures of **2** and **4** are observed (Figs 2 and 3). Two benzimidazole rings in **2** are approximately parallel with the dihedral angle of 13.9(1)°, whereas the dihedral angles between two benzimidazole rings in **4** are 71.8(2)°. This displays that the distorted degree of two benzimidazoles in **4** is larger than that in **2**. The distances between H(1) and H(2) in **2** (3.72 Å) is larger than that (3.32 Å) in **4**.

### Recognition of acetate anion using compound 2 as a sensor

**Figure 4:**
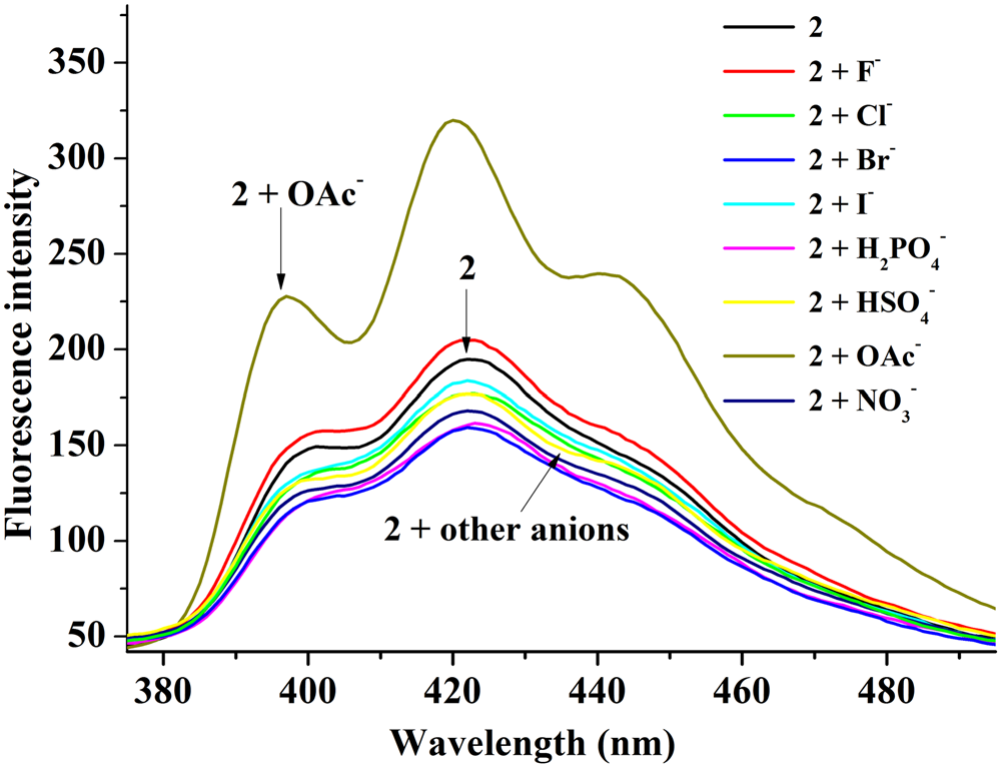
Fluorescence spectra of **2** (1 × 10^−6^ M) upon adding 20 equiv. of salts (H_2_PO_4_^−^, HSO_4_^−^, OAc^−^, NO_3_^−^, I^−^, Br^−^, Cl^−^ and F^−^, and tetrabutyl ammonium being their cations) in H_2_O/CH_3_CN (v:v = 1:1) (*λ*_ex_ = 254 nm, slit: em = 3 nm, ex = 5 nm) at 25 °C.

The recognition of anions (H_2_PO_4_^−^, HSO_4_^−^, OAc^−^, NO_3_^−^, I^−^, Br^−^, Cl^−^ and F^−^) using **2** as a sensor was carried out with the methods of fluorescence and ultraviolet spectroscopy in H_2_O/CH_3_CN (v/v = 1/1) mixed solvent at 25 °C. The compound **2** displayed a wide emission band around 380–495 nm as shown in Fig. 4 (*λ*_ex_ = 254 nm, the slit widths were 5 nm and 3 nm for the excitation and emission). This emission band was attributed to that of anthraquinone. The fluorescence intensity barely changed after adding 20 equiv. of H_2_PO_4_^−^, HSO_4_^−^, NO_3_^−^, I^−^, Br^−^, Cl^−^ and F^−^, respectively, to the solutions of **2**. However, adding 20 equiv. of OAc^−^ caused the remarkable enhancement of fluorescence intensity around 380–495 nm. In Fig. S1, the ultraviolet absorption of **2** at 230–280 nm increased distinctly with the addition of OAc^−^. These experimental results demonstrated that **2** can discriminate effectively OAc^−^ from other anions. Thus, compound **2** may be functioned as a fluorescence chemosensor with high selectivity for OAc^−^.

**Figure 5:**
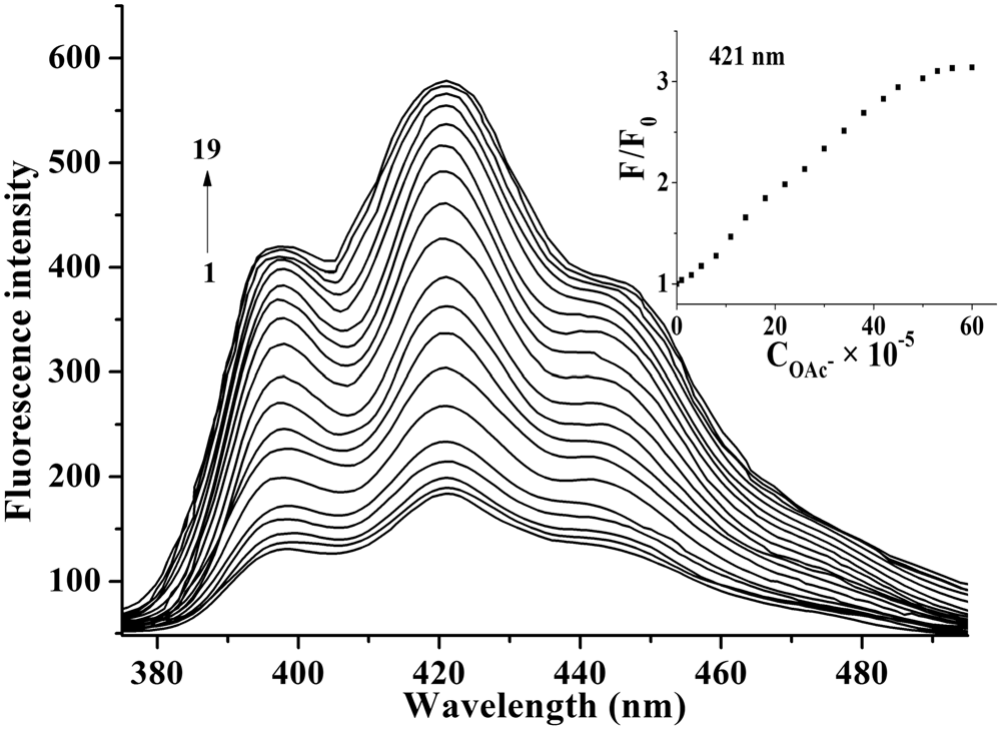
Fluorescent titration curves of **2** (1.0 × 10^−6^ M) in the existence of various concentrations of OAc^−^ in H_2_O/CH_3_CN (v/v = 1/1). C_OAc_− for curves 1–19 are 0, 0.2, 0.5, 1, 1.6, 2.4, 4, 8, 14, 20, 24, 28, 32, 36, 40, 44, 48, 54, 60 × 10^−6^ M (λ_ex_ = 254 nm). Inset: *F*/*F*_0_ of **2** at 421 nm as a function of C_OAc_− (*λ*_ex_ = 254 nm, slit: em = 3 nm, ex = 5 nm) at 25 °C.

As shown in Fig. 5, the fluorescent titration experiments displayed that the fluorescence intensities of **2** around 380–495 nm enhanced gradually along with the incremental concentration of OAc^−^. In the inset of Fig. 5, when the value of C_OAc_^−^/C_**2**_ was between 0 to 44, the fluorescence intensities display rapid increases along with the incremental concentration of OAc^−^. When the value exceeded 44:1, the enhancement of fluorescence intensities slowed down. When the value exceeded 54:1, more OAc^−^ would not cause further rise of fluorescent intensities. The increasing behavior of OAc^−^ about the fluorescence of **2** followed a Stern-Volmer equation (1)^53,54^.

1 $${F}_{0}/F=1+{K}_{SV}{C}_{OA{c}^{-}}$$

in which *F* and *F*_0_ are the fluorescence intensities of **2** with and without OAc^−^, and C_OAc_− is the concentration of OAc^−^. The calculated constant *K*_*SV*_ for **2**·OAc^−^ is 1.2 × 10^4^ M^−1^ (R = 0.996) through employing the equation (1), and the linear parts are in the ranges of 0–44 × 10^−6^ M (Fig. S2). As shown in Fig. S3, the detection limit was determined to be 2.1 × 10^−7^ M according to the changes in OAc^−^ dependent fluorescence intensities^55^.

**Figure 6:**
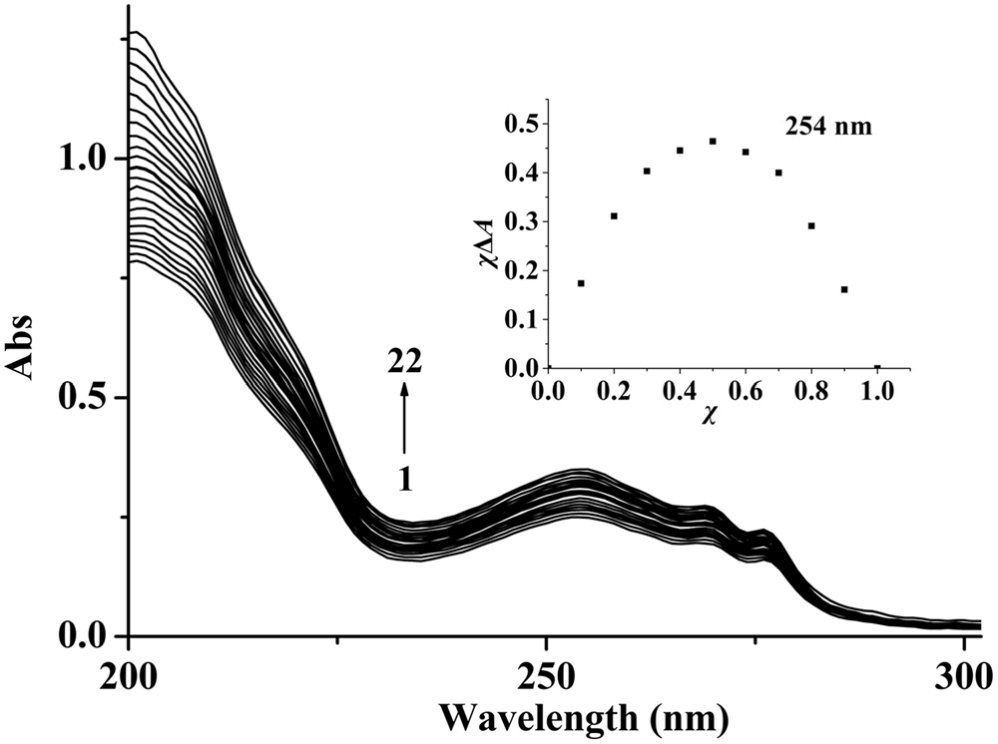
Ultraviolet absorption curves of **2** (1 × 10^−5^ M) in H_2_O/CH_3_CN (v:v = 1:1) at 25 °C. The concentrations of OAc^−^ for curves 1–22 are: 0, 0.4, 0.8, 1.2, 1.6, 3, 4, 6, 9, 12, 15, 18, 21, 24, 27, 30, 33, 38, 43, 48, 54, 60 × 10^−5^ M. Inset: the Job’s plot for a **2**·OAc^−^ complex at 254 nm. *χ* is molar fractions of **2**, and *χ*Δ*A* is the products between molar fractions and the discrepancy of the absorption bands.

In ultraviolet titration experiments (Fig. 6), the ultraviolet absorption of **2** enhanced gradually along with the increasing concentration of OAc^−^. The stability constant (*K*_*S*_) was calculated according to the following relation of non-linear least square analysis of the titration curves for 1:1 complexation^56^.

2 $${\rm{\Delta }}A={\rm{B}}\{{{\rm{C}}}_{{\bf{2}}}+{{\rm{C}}}_{{{\rm{OAc}}}^{-}}+1/{{\rm{K}}}_{{{\rm{S}}}^{-}}{[{({{\rm{C}}}_{{\bf{2}}}+{{\rm{C}}}_{{{\rm{OAc}}}^{-}}+1/{{\rm{K}}}_{{\rm{S}}})}^{2}-4{{\rm{C}}}_{{\bf{2}}}{{\rm{C}}}_{{{\rm{OAc}}}^{-}}]}^{1/2}\}$$

where Δ*A* is the discrepancy between the absorbance with or without OAc^−^ (Δ*A* = *A* − *A*_0_), B is a floating parameter, C_OAc_− and C_**2**_ are the concentrations of guest and host.

As displayed in Fig. S4, the plot of Δ*A* versus C_OAc_− showed good non-linear relationship for OAc^−^, which indicates the formation of 1:1 complexation between **2** and OAc^−^. The stability constant *K*_*S*_ was 2.3 × 10^4^ M^−1^ (R = 0.991). Noteworthy, Job’s plot at 254 nm also further demonstrated to form a 1:1 complexation for **2**·OAc^−^ (Inset of Fig. 6)^57–59^. In the recognition of OAc^−^ using **2** as a host, the *K*_*SV*_ value (1.2 × 10^4^ M^−1^) from the fluorescence method and the *K*_*S*_ value (2.3 × 10^4^ M^−1^) from the ultraviolet method are consistent with each other^60^. Compared to the literatures, the binding constants of **2** to OAc^−^ are in the middle of the values of literatures reported, and the detection limit of **2** to OAc^−^ is close to the minimum in literatures reported (the binding constants and detection limits of these reports being in the range of 6.9 × 10^2^ M^−1^ to 5.9 × 10^5^ M^−1^ and 1.2 × 10^−7^ mol/L to 1.0 × 10^−6^ mol/L)^10,11,19–21,23,24,26^.

In competition experiments, 20 equiv. of other anions (H_2_PO_4_^−^, HSO_4_^−^, NO_3_^−^, I^−^, Br^−^, Cl^−^ and F^−^) was mixed with **2** (1 × 10^−6^ M), and then 20 equiv. of OAc^−^ was added. The fluorescence spectra show that there is no obvious enhancement or decrease in the existence of 20 equiv. of interfering anions (Fig. S5). The results show that **2** has the immunity from interference of other anions.

### Interactions of acetate anion with 2

**Figure 7:**
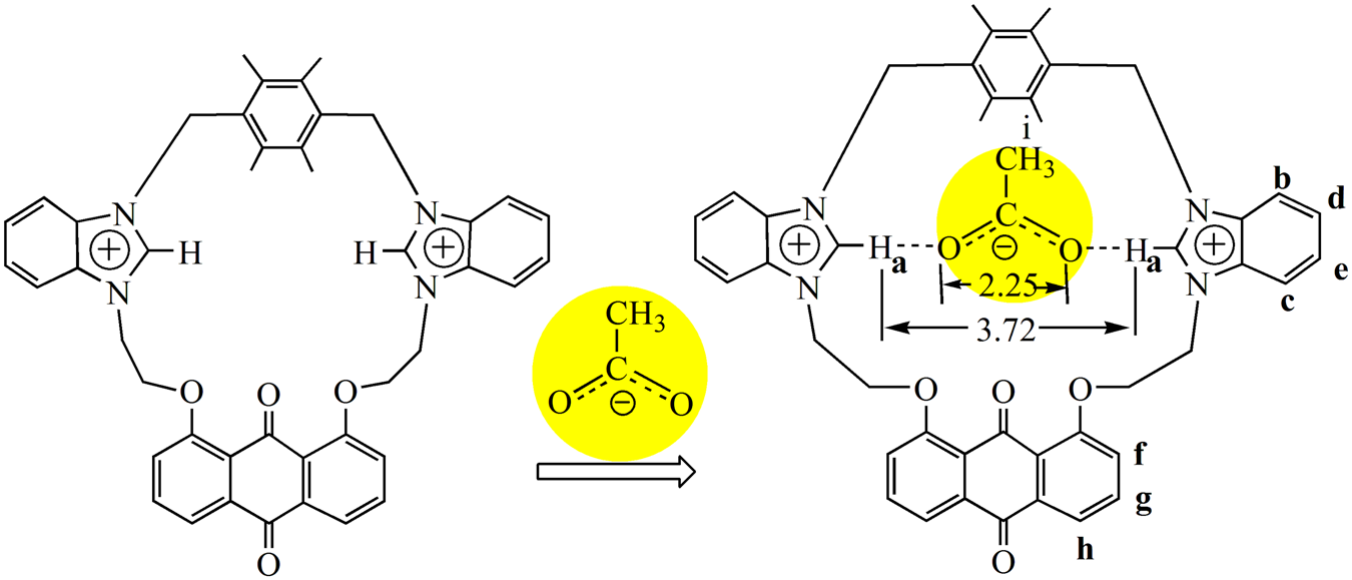
The interactions of OAc^−^ with **2**.

**Figure 8:**
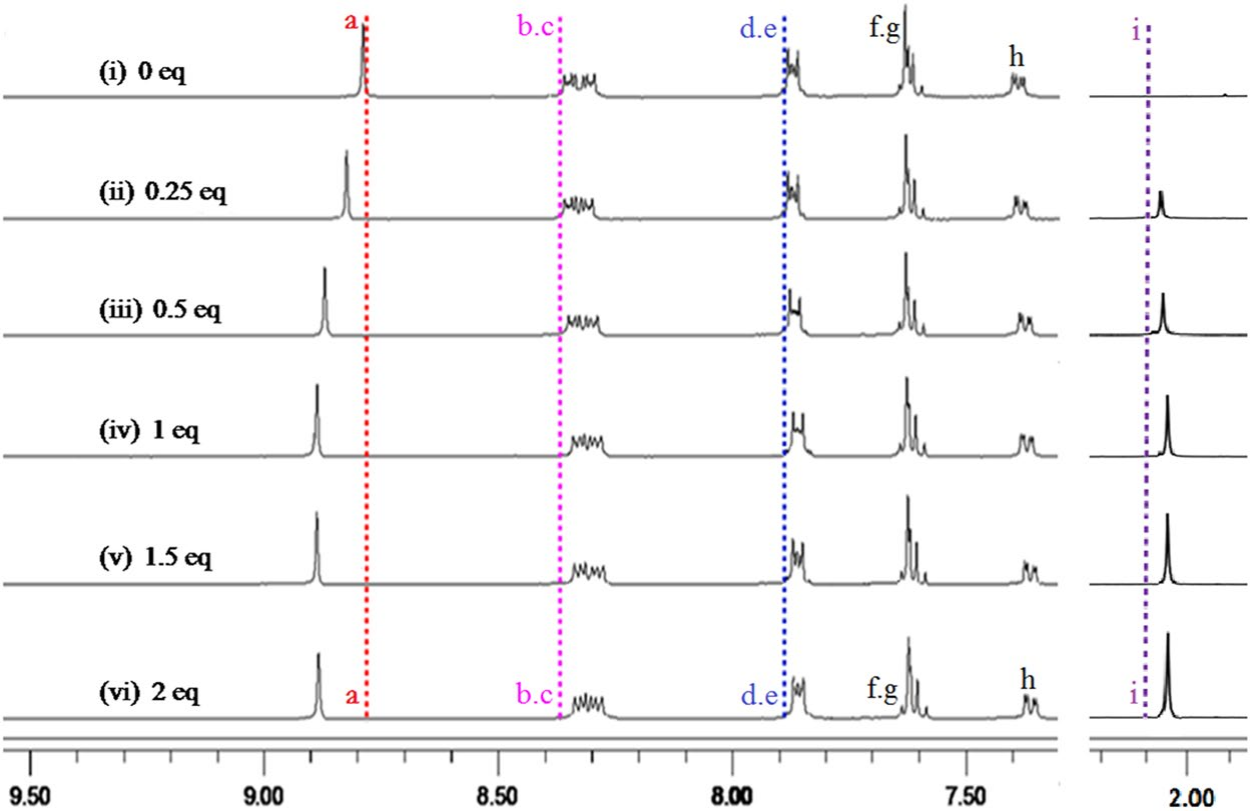
Partial ^1^H NMR spectra. (i) **2**; (ii) **2** and 0.25 equivalent of OAc^−^; (iii) **2** and 0.5 equivalent of OAc^−^; (iv) **2** and 1 equivalent of OAc^−^; (v) **2** and 1.5 equivalent of OAc^−^; (vi) **2** and 2 equivalent of OAc^−^.

Through analyzing the cavity size and structure characteristics of **2** (*H*(1)···*H*(2) separation being 3.72 Å in Fig. 2), the size of OAc^−^ (the distance of two oxygen atoms in OAc^−^ being 2.25 Å)^61^ is suitable with that of **2**. As shown in Fig. 7, *H*a of host **2** is the most possible binding site for OAc^−^ and the acting force between **2** and OAc^−^ may be C-H···O hydrogen bonds. To gain related information about the binding mode of **2** and OAc^−^, ^1^H NMR titration experiments was performed in DMSO-*d*_6_ (Fig. 8). As displayed in Fig. 8(iv), the chemical shifts of *H*a shifted downfield by 0.10 ppm, and the chemical shifts of *H*b-*H*e shifted upfield by 0.02–0.03 ppm in the presence of 1 equiv. of OAc^−^. Additionally, the chemical shifts of *H*f decreased by 0.03 ppm. These results indicated there existed C-H···O hydrogen bonds between **2** and OAc^−^. By comparison of Fig. 8(v,vi), chemical shifts of protons (*H*a-*H*f) did not have further change in the presence of more OAc^−^, which proved the formation of 1:1 complexation between **2** and OAc^−^. In HRMS of **2**·OAc^−^ (Fig. S14), m/z (747.29) of [**2** − 2(PF_6_^−^) + OAc^−^]^+^ was observed, which further confirmed that a 1:1 complexation between **2** and OAc^−^ was formed. All these results accorded with the survey of the Job’s plot experiment (Fig. 5). To further know the complexation property of **2** with OAc^−^, IR spectra of free **2**, OAc^−^ and **2**·OAc^−^ were measured (Fig. S16). The C-H flexural vibration bands moved from 841 cm^−1^ in free **2** to 837 cm^−1^ in **2**·OAc^−^, and the C-H stretching vibration moved from 2959 cm^−1^ in free OAc^−^ to 2962 cm^−1^ in **2**·OAc^−^. The C=O bands of OAc^−^ moved from 1677 cm^−1^ in OAc^−^ to 1667 cm^−1^ in **2**·OAc^−^. The C-N bands moved from 1587 cm^−1^ in free **2** to 1584 cm^−1^ in **2**·OAc^−^.

Through the comprehensive analysis of ^1^H NMR titrations, HRMS spectra, IR spectra and structure of **2**, the binding force between **2** and OAc^−^ is mainly attributed to C-H···O hydrogen bonds because of strong affinity of hydrogen atom toward oxygen atom. Upon the combination of **2** and OAc^−^, the fluorescence intensity of **2** enhanced remarkably. We tried to the cultivation of single crystal for **2**·OAc^−^ to determine its solid-state structure. Unfortunately, no single crystals can be gotten.

### Recognition of nitrate anion using 4 as a sensor

The selective recognition of some anions (H_2_PO_4_^−^, HSO_4_^−^, OAc^−^, NO_3_^−^, I^−^, Br^−^, Cl^−^ and F^−^, and their cations being tetrabutyl ammonium (TBA^+^)) using **4** as a sensor was also investigated by using analogous methods of **2** for OAc^−^. The results showed that **4** was a highly selective fluorescent sensor for NO_3_^−^, and it can discriminate between NO_3_^−^ and other anions (Figs S6–S13). The Stern-Volmer constant *K*_*SV*_ value was calculated as 1.5 × 10^4^ M^−1^ (R = 0.998) for **4**·NO_3_^−^ based on fluorescence method (Fig. S9), and it was similar to the stability constant *K*_*S*_ value (2.5 × 10^4^ M^−1^) from the UV/vis method (Fig. S12)^60^. The detection limit (2.6 × 10^−7^ M) was estimated from the changes in NO_3_^−^ dependent fluorescence intensity (Fig. S10), and this value is in the middle of the values of the literatures (the detection limits of these reports are in the range of 4.7 × 10^−7^–1.7 × 10^−8^ mol/L)^22,25,27^.

**Figure 9:**
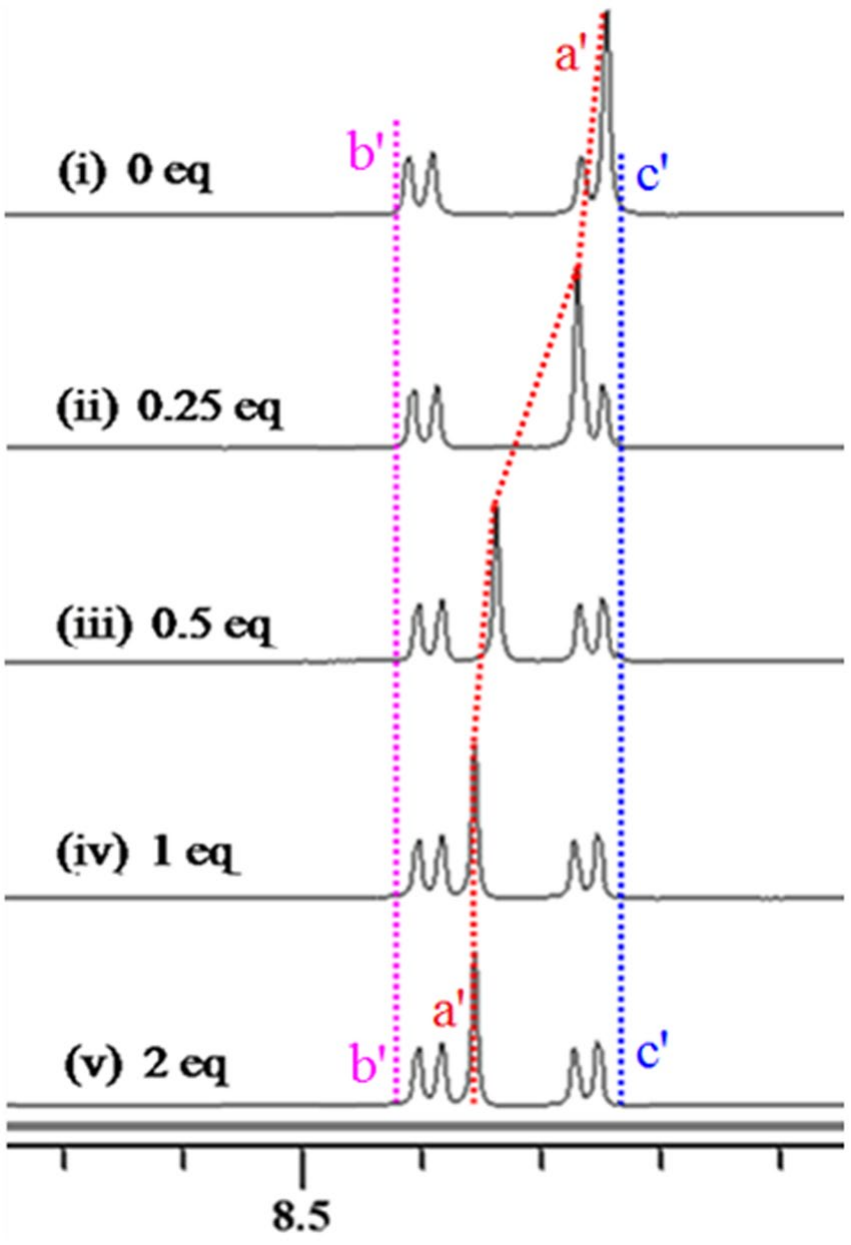
Partial ^1^H NMR spectra in DMSO-*d*_6_. (i) **4**; (ii) **4** and 0.25 equivalent of NO_3_^−^; (iii) **4** and 0.5 equivalent of NO_3_^−^; (iv) **4** and 1 equivalent of NO_3_^−^; (v) **4** and 2 equivalent of NO_3_^−^.

**Figure 10:**
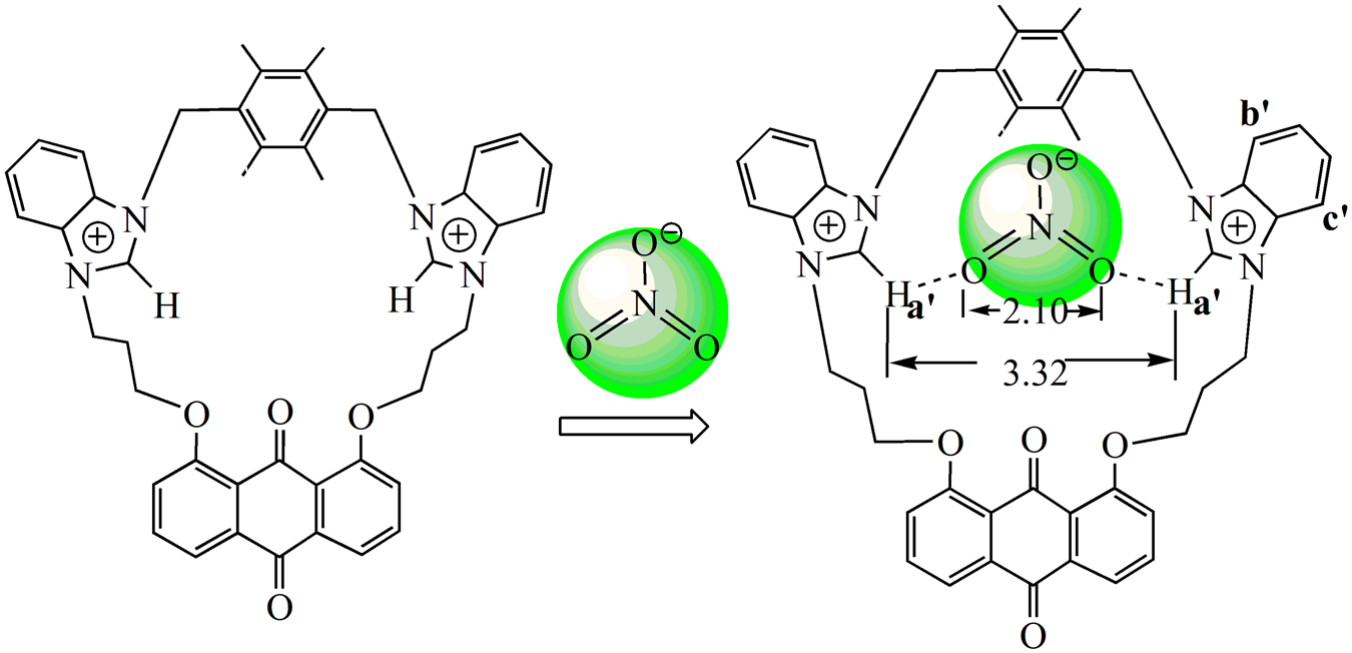
The interactions of NO_3_^−^ with **4**.

The spectral differences in ^1^H NMR titration experiments of **4** are depicted in Fig. 9. *H*a’ has a large shift (0.12 ppm) with the addition of 1 equiv. of NO_3_^−^ as shown in Fig. 9(iv). At the same time, the signals of *H*b’ and *H*c’ shifted upfield by 0.02 ppm upon addition of 1 equiv. of NO_3_^−^. These changes should be attributed to the formation of C-H···O hydrogen bonds between NO_3_^−^ and C(2)-H of benzimidazolium (Fig. 10). In addition, the chemical shifts of the other protons in **4** do not obviously change upon the addition of more equivalents of NO_3_^−^ (Fig. 9(v)). This indicates that a 1:1 complex between **4** and NO_3_^−^ was formed. In high resolution mass spectrometry (HRMS) analysis of **4**·NO_3_^−^ (Fig. S15), m/z (778.30) of [**4** − 2(PF_6_^−^) + NO_3_^−^]^+^ is observed, which provides additional evidence for the formation of a 1:1 complex between **4** and NO_3_^−^. These results are consistent with the result of the Job’s plot experiment (Inset of Fig. S11).

To further understand the complexation behavior of **4** with NO_3_^−^, the infrared spectra (IR) of **4**, NO_3_^−^ and **4**·NO_3_^−^ were measured. In the infrared spectra (Fig. S17), we observed that N=O absorption bands of NO_3_^−^ move from 1338 cm^−1^ in NO_3_^−^ to 1347 cm^−1^ in **4**·NO_3_^−^, C-H flexural vibration absorption bands move from 747 cm^−1^ in free **4** to 751 cm^−1^ in **4**·NO_3_^−^, and the C-H stretching vibration in free **4** move from 2954 cm^−1^ in free **4** to 2963 cm^−1^ in **4**·NO_3_^−^. The C-N flexural vibration absorption bands move from 1583 cm^−1^ in free **4** to 1589 cm^−1^ in **4**·NO_3_^−^.

### Applications of 2 or 4 in real samples

The practical applications of **2** or **4** were estimated through the determination of OAc^−^ or NO_3_^−^ added in real water samples (tap water and drinking water). The water samples were mixed with known concentrations of OAc^−^ (8.0 × 10^−6^ mol/L) or NO_3_^−^ (15.0 × 10^−6^ mol/L) and analyzed by the standard methods. Each sample was analyzed with their three replicates. The results were summarized in Tables 1 and 2, and these results had small relative standard deviation (RSD) values. Therefore, the sensors **2** or **4** have potential application ability for OAc^−^ or NO_3_^−^ in real samples.

**Table 1:** Application of **2** in real samples.

Sample	OAc^−^ added (μM)	OAc^−^ found (μM)	RSD^a^ (%)
Tap water	8.00	8.20	2.57
Drinking water	8.00	7.80	1.61

^a^Relative standard deviation of three individual measurements.

**Table 2:** Application of **4** in real samples.

Sample	NO_3_^−^ added (μM)	NO_3_^−^ found (μM)	RSD^a^ (%)
Tap water	15.00	15.10	2.05
Drinking water	15.00	14.70	1.86

^a^Relative standard deviation of three individual measurements.

## Conclusion

In summary, two new 23-membered and 25-membered macrocyclic compounds **2** and **4** with bis-benzimidazolium groups have been synthesized and characterized. The anion recognition abilities of **2** or **4** have been investigated. The fluorescence and ultraviolet titrations show that **2** can detect effectively acetate anion. The *K*_*SV*_ value (1.2 × 10^4^ M^−1^) and *K*_*S*_ value (2.3 × 10^4^ M^−1^) for **2**·OAc^−^ are similar to each other. Even if the detection limit is down to 2.1 × 10^−7^ mol/L, the detection of **2** to OAc^−^ is sensitive. Analogously, **4** has special selectivity for nitrate anion. The *K*_*SV*_ value (1.5 × 10^4^ M^−1^) and *K*_*S*_ value (2.5 × 10^4^ M^−1^) for **4**·NO_3_^−^ are too similar to each other. Even if the detection limit is down to 2.6 × 10^−7^ mol/L, the detection of **4** to NO_3_^−^ is sensitive. The differences between **2** and **4** in the course of anion recognitions are mainly related to the structural characteristics of the macrocycles. The distance between two C(2)-H of benzimidazolium in **2** (3.72 Å) is larger than that in **4** (3.32 Å), therefore, **2** can effectively match with OAc^−^, and **4** can match effectively with NO_3_^−^ (the distance between two oxygen atoms in OAc^−^ (2.25 Å) being larger than in NO_3_^−^ (2.10 Å)). The experiment results reveal that compounds **2** and **4** have good application prospects in anion recognitions. Our ongoing research is aiming at recognizing anions more efficiently, and the development of some environment-friendly, highly selective and highly sensitive chemosensors are underway.

## Experimental

### General procedures

Total commercially available chemicals for synthesis and test were of reagent grade. A Boetius Block apparatus was used for the report of melting points. A PerkinElmer Spectrum 100 FT-IR spectrophotometer was used for the report of Infrared (IR) spectra. A Varian spectrometers was used for the report of ^1^H NMR and ^13^C NMR spectra. The measurement of the elemental analyses was carried out on a Perkin-Elmer 2400C Elemental Analyzer. Ultraviolet spectra were recorded on a PerkinElmer Lamber35 UV spectrophotometer. The fluorescence spectra were carried out in a Shimadzu RF-5301PC fluorescence spectrophotometer. A VG ZAB-HS mass spectrometer was used to record EI mass spectra.

### 1,4-Di(benzimidazole-methyl)-durene

10 mL of HBr/acetic acid solution (31 wt%) was added to the glacial acetic acid (30 mL) solution of durene (6.710 g, 50.0 mmol) and paraformaldehyde (3.075 g, 102.5 mmol). The mixture was heated at 120 °C for 8 hours, and then this mixture was poured into 50 mL of H_2_O to precipitate a white powder, which was collected through filtration to give 1,4-di(bromomethyl)-durene. Yield: 15.205 g (95%). M.p.: 192–194 °C.

TBAB (0.260 g, 0.8 mmol), KOH (2.000 g, 35.6 mmol) and benzimidazole (2.150 g, 18.2 mmol) were added to 100 mL of CH_3_CN, and this suspension was stirred under refluxing for 1 hour. To above mixture was dropwise added a CH_3_CN (50 mL) solution of 1,4-di(bromomethyl)-durene (2.880 g, 9.0 mmol), and the reaction was carried out at 80 °C for 72 hours. After CH_3_CN was removed via rotary evaporation, CH_2_Cl_2_ (100 mL) was added to the residue, which was washed with water (3 × 100 mL). The CH_2_Cl_2_ solution was dried over anhydrous MgSO_4_. After CH_2_Cl_2_ was removed via rotary evaporation, a white powder of 1,4-di(benzimidazol-1-yl-methyl)-durene was gotten. Yield: 3.014 g (86%). Anal. Calcd for C_26_H_26_N_4_: C, 79.14; H, 6.65; N, 14.21%. Found: C, 79.32; H, 6.79; N, 14.42%. M.p.: 238–240 °C. ^1^H NMR (400 MHz, DMSO-*d*_6_): *δ* 2.21 (s, 12H, C*H*_3_), 5.51 (s, 4H, C*H*_2_), 7.31 (m, 4H, Ar*H*), 7.63 (d, *J* = 7.6 Hz, 2H, Ph*H*), 7.68 (d, *J* = 7.6 Hz, 2H, Ar*H*), 7.72 (s, 2H, 2-benzim*H*) (benzim = benzimidazole).

### 1,8-Di(2′-bromoethoxy)-9,10-anthraquinone

1,2-Dibromoethane (4.691 g, 24.9 mmol) was dropwise added to a suspension of anhydrous K_2_CO_3_ (3.446 g, 24.8 mmol), 1,8-dihydroxy-9,10-anthraquinone (1.082 1.000 g, 4.1 mmol), TBAB (0.184 g, 0.5 mmol) in acetone (50 mL), and the mixture was stirred under refluxing for 72 hours. After acetone was removed, 60 mL of water was added to the residue. The aqueous was extracted with CH_2_Cl_2_ (3 × 30 mL) and the combined solvent was removed via rotary evaporation after drying over anhydrous MgSO_4_. A yellow powder of 1,8-di(2′-bromoethoxy)-9,10-anthraquinone was gotten. Yield: 1.133 g (60%). Anal. Calcd for C_18_H_14_Br_2_O_4_: C, 47.61; H, 3.11%. Found: C, 47.84; H, 3.44%. M.p.: 122–124 °C. ^1^H NMR (400 MHz, DMSO-*d*_6_): *δ* 3.86 (t, *J* = 5.8 Hz, 4H, C*H*_2_), 4.50 (t, *J* = 5.8 Hz, 4H, C*H*_2_), 7.58 (q, *J* = 3.2 Hz, 2H, Ar*H*), 7.76 (t, *J* = 2.8 Hz, 4H, Ar*H*). ^13^C NMR (100 MHz, DMSO-*d*_6_): *δ* 182.8 (*C*=O), 180.7 (*C*=O), 157.3 (Ar*C*), 134.2 (Ar*C*), 134.1 (Ar*C*), 124.2 (Ar*C*), 121.5 (Ar*C*), 119.2 (Ar*C*), 69.7 (O*C*H_2_CH_2_), 30.8 (OCH_2_*C*H_2_).

### 1,8-Bis(3′-bromopropoxy)-9,10-anthraquinone

This compound was prepared in a manner analogous to that for 1,8-bis(2′-bromoethoxy)-9,10-anthraquinone, only 1,3-dibromopropane (5.450 g, 27.0 mmol) was used instead of 1,2-dibromoethane. Yield: 1.735 g (80%). M.p.: 145–147 °C. Anal. Calcd for C_20_H_18_Br_2_O_4_: C, 49.82; H, 3.76%. Found: C, 49.83; H, 3.84%. ^1^H NMR (400 MH_Z_, DMSO-*d*_6_): *δ* 2.10 (m, 4H, C*H*_2_), 3.65 (t, *J* = 6.6 Hz, 4H, C*H*_2_), 4.01 (t, *J* = 5.6 Hz, 4H, C*H*_2_), 7.33 (t, *J* = 4.2 Hz, 2H, Ar*H*), 7.53 (m, 4H, Ar*H*). ^13^C NMR (100 MH_Z_, DMSO-d_6_): *δ* 183.5 (*C*=O), 181.9 (*C*=O), 157.5 (Ar*C*), 141.8 (Ar*C*), 135.4 (Ar*C*), 134.3 (Ar*C*), 132.4 (Ar*C*), 131.6 (Ar*C*), 130.9 (Ar*C*), 127.5 (Ar*C*), 127.4 (Ar*C*), 123.8 (Ar*C*), 114.40 (Ar*C*), 45.2 (*C*H_2_), 31.2 (*C*H_2_), 16.3 (*C*H_2_).

### Macrocyclic Compound 2

To a CH_3_CN (200 mL) solution of 1,8-di(2′-bromoethoxy)-9,10-anthraquinone (0.455 g, 1.0 mmol) was added a CH_3_CN (200 mL) solution of 1,4-di(benzimidazol-1-yl-methyl)-durene (0.394 g, 1.0 mmol). After refluxing for 48 hours, a yellow solid was generated. The solid was collected through filtration to afford macrocyclic compound **1** with two bromide anions.

A solution of NH_4_PF_6_ (0.391 g, 2.3 mmol) in methanol (20 mL) was added to a solution of compound **1** (0.878 g, 1.0 mmol) in methanol (40 mL), and then the mixed solution was stirred for 2.5 days at 25 °C to precipitate a pale yellow solid. The solid was collected through filtration to give macrocyclic compound **2** with two hexafluorophosphate anions. M.p.: 282–284 °C. Yield: 0.723 g (74%). Anal. Calcd for C_44_H_40_F_12_N_4_O_4_P_2_: C, 53.99; H, 4.11; N, 5.72%. Found: C, 53.87; H, 4.23; N, 5.65%. ^1^H NMR (400 MHz, DMSO-*d*_6_): *δ* 2.05 (s, 12H, C*H*_3_), 4.51 (d, *J* = 3.6 Hz, 4H, OCH_2_C*H*_2_), 5.05 (d, *J* = 4.0 Hz, 4H, OC*H*_2_CH_2_), 5.78 (s, 4H, C*H*_2_), 7.40 (q, *J* = 3.2 Hz, 2H, Ar*H*), 7.62 (t, *J* = 3.2 Hz, 4H, Ar*H*), 7.87 (q, *J* = 3.2 Hz, 4H, Ar*H*), 8.36 (m, 4H, Ar*H*), 8.82 (s, 2H, 2-benzim*H*). ^13^C NMR (100 MHz, DMSO-*d*_6_): *δ* 183.4 (*C*=O), 182.2 (*C*=O), 156.1 (Ar*C*), 142.6 (Ar*C*), 134.9 (Ar*C*), 134.5 (Ar*C*), 131.1 (Ar*C*), 126.2 (Ar*C*), 121.7 (Ar*C*), 120.3 (Ar*C*), 119.0 (Ar*C*), 113.5 (Ar*C*), 67.8 (O*C*H_2_CH_2_), 44.7 (N*C*H_2_), 30.6 (N*C*H_2_), 16.3 (*C*H_3_), 15.8 (*C*H_3_).

### Macrocyclic Compound 4

This compound was prepared in a manner analogous to that for compound **2**, only 1,8-bis(3’-bromopropoxy)-9,10-anthraquinone (0.482 g, 1.0 mmol) was used instead of 1,8-bis(2′-bromoethoxy)-9,10-anthraquinone. Yield: 0.795 g (79%). M.p.: 286–288 °C. Anal. Calcd for C_46_H_44_F_12_N_4_O_4_P_2_: C, 54.87; H, 4.40; N, 5.56%. Found: C, 54.74; H, 4.52; N, 5.63%. ^1^H NMR (400 MHz, DMSO-*d*_6_): *δ* 1.85 (s, 12H, C*H*_3_), 2.49 (d, *J* = 1.6 Hz, 4H, OCH_2_C*H*_2_CH_2_), 3.93 (t, *J* = 5.4 Hz, 4H, OCH_2_CH_2_C*H*_2_), 4.80 (t, *J* = 6.4 Hz, 4H, OC*H*_2_CH_2_CH_2_), 5.71 (s, 4H, C*H*_2_), 7.40 (q, *J* = 3.2 Hz, 2H, Ar*H*), 7.85 (m, 8H, Ar*H*), 8.26 (d, *J* = 8.4 Hz, 4H, Ar*H*), 8.41 (d, *J* = 8.0 Hz, 2H, 2-benzim*H*). ^13^C NMR (100 MHz, DMSO-*d*_6_): *δ* 183.5 (*C*=O), 182.4 (*C*=O), 157.5 (Ph*C*), 141.8 (Ar*C*), 135.4 (Ar*C*), 134.3 (Ar*C*), 132.4 (Ar*C*), 131.6 (Ar*C*), 130.9 (Ar*C*), 127.5 (Ar*C*), 123.8 (Ar*C*), 120.7 (Ar*C*), 119.3 (Ar*C*), 114.4 (Ar*C*), 69.0 (O*C*H_2_CH_2_), 53.2 (N*C*H_2_), 45.1 (N*C*H_2_), 31.1 (CH_3_*C*H_2_CH_3_), 26.1 (*C*H_3_), 16.2 (*C*H_3_).

### Fluorescence Titrations

The stock solution (1.0 × 10^−4^ M) of the host was prepared and diluted to the suitable concentration with H_2_O/CH_3_CN (v:v = 1:1). The stock solutions (1.0 × 10^−4^ M or 1.0 × 10^−3^ M) of guest were prepared and diluted in the same solvent. Test solutions were prepared through placing 0.1 mL of host stock solution into a 10 mL volumetric flask, and the appropriate amount of the stock solutions (1.0 × 10^−4^ M or 1.0 × 10^−3^ M) of guest were added with a microsyringe. The mixture solutions were diluted to 10 mL with H_2_O/CH_3_CN (1:1) to prepare test solutions. The concentrations of guest in the test solutions were from 0 to 60.0 × 10^−6^ M, and the concentration of host stayed the same (1.0 × 10^−6^ M). The test solutions were kept at 25 °C for 10 minutes, and then fluorescence spectra were recorded with the excitation wavelength at 254 nm. Stern-Volmer constant *K*_*SV*_ was derived from plots of *F*_0_/*F* vs C_OAc_− × 10^−6^ (or C_NO3_− × 10^−6^) using Origin 8.0.

### Job’s plot

The stock solution (1.0 × 10^−4^ M) of the host was prepared and diluted to the suitable concentration with H_2_O/CH_3_CN (v:v = 1:1). The stock solutions (1.0 × 10^−4^ M or 1.0 × 10^−3^ M) of tetrabutylammonium salts of H_2_PO_4_^−^, HSO_4_^−^, OAc^−^, NO_3_^−^, I^−^, Br^−^, Cl^−^ and F^−^ were prepared and diluted in the same solvent. Test solutions were prepared through placing 1 mL of host stock solution into a volumetric flask of 10 mL, and the appropriate amount of the stock solutions (1.0 × 10^−4^ M or 1.0 × 10^−3^ M) of tetrabutylammonium salts were added with a microsyringe. The mixture solutions were diluted to 10 mL with H_2_O/CH_3_CN (1:1) to prepare test solutions. The molar fractions of host and anion in the test solutions were from 1 to 0 and 0 to 1, respectively. The total concentration is 1.0 × 10^−5^ M. The test solutions were kept at 25 °C for 10 minutes, and then absoption spectra were measured.

### Ultraviolet titrations

According to the methods in fluorescence titrations, the stock solutions and test solutions of UV/vis titrations were prepared. The concentrations of guest in the test solutions were from 0 to 60.0 × 10^−5^ M, and the concentration of host stayed the same (1.0 × 10^−5^ M). The test solutions were kept at 25 °C for 10 minutes, and then absoption spectra were measured.

### Real sample analysis

The stock solution (1.0 × 10^−4^ M) of the host was prepared and diluted to the suitable concentration with H_2_O/CH_3_CN (v:v = 1:1). The stock solutions (1.0 × 10^−4^ M or 1.0 × 10^−3^ M) of guest were prepared and diluted in the same solvent. The tap water and drinking water were obtained from the college of chemistry, Tianjin Normal University. Different anions were added in the real water samples. Test solutions were prepared according to the methods in fluorescence titrations. The concentrations of OAc^−^ or NO_3_^−^ were 8.0 × 10^−6^ M and 15.0 × 10^−6^ M, respectively. The test solutions were kept at 25 °C for 10 minutes, and then fluorescence spectra were recorded with the excitation wavelength at 254 nm.

### X-ray structure determinations

A Bruker Apex II CCD diffractometer were used for the collection of diffraction data of **2** and **4**^62^. The structure was solved with the SHELXS program^63^. Figures 1 and 2 were formed via employing Crystal-Maker^64^. Other details for structural analysis and crystallographic data was listed in Table S1.

## Supplementary information

Supplementary Information

## References

[1] Yao H (2018). A novel supramolecular AIE gel acts as a multi-analyte sensor array. New J. Chem..

[2] Santos-Figueroa LE (2013). Chromogenic and fluorogenic chemosensors and reagents for anions. A comprehensive review of the years 2010–2011. Chem. Soc. Rev..

[3] Shumilova TA, Rüffer T, Lang H, Kataev EA (2018). Straightforward design of fluorescent receptors for sulfate: Study of non-covalent interactions contributing to host-guest formation. Chem. Eur. J..

[4] Lu QS (2009). Imidazolium-functionalized BINOL as a multifunctional receptor for chromogenic and chiral anion recognition. Org. Lett..

[5] Akcil A (2003). Destruction of cyanide in gold mill effluents: Biological versus chemical treatments. Biotech. Adv..

[6] Pearce LL, Bominaar EL, Hill BC, Peterson J (2003). Reversal of cyanide inhibition of cytochrome *c* oxidase by the auxiliary substrate nitric oxide. J. Biol. Chem..

[7] Cummings TF (2004). The treatment of cyanide poisoning. Occ. Med..

[8] Manez RM, Sancenon F (2003). Fluorogenic and chromogenic chemosensors and reagents for anions. Chem. Rev..

[9] Nishiyabu R, Anzenbacher P (2006). 1,3-Indane-based chromogenic calixpyrroles with push-pull chromophores: Synthesis and anion sensing. Org. Lett..

[10] Xu K (2015). Novel anthracene-based fluorescent sensor for selective recognition of acetate anions in protic media. Spectrochimi Acta A.

[11] Singh A, Tom S, Trivedi DR (2018). Aminophenol based colorimetric chemosensor for naked-eye detection of biologically important fluoride and acetate ions in organo-aqueous medium: Effective and simple anion sensors. J. Photoch. Photobio. A.

[12] Manju S, Jose L, Gopal TKS, Ravishankar CN, Lalitha KV (2007). Effects of sodium acetate dip treatment and vacuum-packaging on chemical, microbiological, textural and sensory changes of pearlspot (Etroplus suratensis) during chill storage. Food Chem..

[13] Sallam KI (2007). Antimicrobial and antioxidant effects of sodium acetate, sodium lactate, and sodium citrate in refrigerated sliced salmon. Food control.

[14] Bruning-Fann CS, Kaneene JB (1993). The effects of nitrate, nitrite and N-nitroso compounds on human health: a review. Vet. Hum. Toxicol..

[15] Knobeloch L, Salna B, Hogan A, Postle J, Anderson H (2000). Blue babies and nitrate-contaminated well water. Environ. Health Perspect..

[16] Ke GH, Wang P, Yang Z (2006). Plant uptake of non-ionic organic chemicals. Environ. Sci. Technol..

[17] Butler A (2015). Nitrites and nitrates in the human diet: Carcinogens or beneficial hypotensive agents. J. Ethnopharmacology.

[18] Chetty AA, Prasad S (2009). Flow injection analysis of nitrate-N determination in root vegetables: Study of the effects of cooking. Food Chem..

[19] Pandian TS, Srinivasadesikan V, Lin MC, Kang JA (2015). selective acetate anion binding receptor: Participation via cationic CH_3_ donors. Tetrahedron.

[20] Guadalupe Hernandez J, Huerta-Aguilar CA, Thangarasu P, Hopfl H (2017). A ruthenium(III) complex derived from N,N′-bis(salicylidene)ethylenediamine as a chemosensor for the selective recognition of acetate and its interaction with cells for bio-imaging: Experimental and theoretical studies. New J. Chem..

[21] Huang WW, Li YP, Lin H, Lin H (2012). Colorimetric recognition of acetate anions in aqueous solution using charge neutral azo derivatives. Spectrochimi. Acta A.

[22] Chen SY, Ni XL (2016). Development of an AIE based fluorescent probe for the detection of nitrate anions in aqueous solution over a wide pH range. RSC Adv..

[23] Singh A, Trivedi DR (2017). ‘Naked-eye’ detection of biologically important anions in aqueous media by colorimetric receptor and its real life applications. Spectrochimi Acta A.

[24] Sekutor M, Opacak S, Aleskovic M, Mlinaric-Majerski K (2015). Synthesis and anion binding properties of a novel 1,8-dipyrrolecarbazole Schiff base. Croat. Chem. Acta.

[25] Tan JY (2018). Chalcone based ion-pair recognition towards nitrates and the application for the colorimetric and fluorescence turn-on determination of water content in organic solvents. Sensor. Actuat. B-Chem..

[26] Qasem RJ, Farh IK, Essa M (2017). A novel LC-MS/MS method for the quantitative measurement of the acetate content in pharmaceutical peptides. J. Pharmaceut. Bio. Anal..

[27] Nemade K, Fegade U, Attarde S, Ingle S (2015). Determination of nitrite and nitrate in water and leafy vegetable samples using ion chromatography with conductivity detection. Chem. Sci. Rev. Lett..

[28] Hu YM, Wang XM, Fei D, Dong FQ, Ding LS (2008). Study on the interaction between Riboflavin and herring sperm DNA by fluorescence spectrometry. Acta Chim. Sinica.

[29] Wang CC (2014). A ratiometric fluorescent chemosensor for Hg^2+^ based on FRET and its application in living cells. Sensor. Actuat. B-Chem..

[30] Morteza H (2012). Selective recognition of acetate ion based on fluorescence enhancement chemosensor. Luminescence.

[31] Choi K, Hamilton AD (2001). Selective anion binding by a macrocycle with convergent hydrogen bonding functionality. J. Am. Chem. Soc..

[32] Caballero A (2014). Discovery of anion-π interactions in the recognition mechanism of inorganic anions by 1,2,3-triazolium rings. Chem. Commun..

[33] Guha S, Saha S (2010). Fluoride ion sensing by an anion-π interaction. J. Am. Chem. Soc..

[34] Barryman OB, Sather AC, Hay BP, Meisner JS, Johnson DW (2008). Solution phase measurement of both weak *σ* and C-H···X^−^ hydrogen bonding interactions in synthetic anion receptors. J. Am. Chem. Soc..

[35] Chifotides HT, Schottel BL, Dunbar KR (2010). The π-accepting arene HAT(CN)_6_ as a halide receptor through charge transfer: Multisite anion interactions and self-assembly in solution and the solid state. Angew. Chem., Int. Ed..

[36] Rosokha YS, Lindeman SV, Rosokha SV, Kochi JK (2004). Halide recognition through diagnostic “anion-π“ interactions: Molecular complexes of Cl^−^, Br^−^, and I^−^ with olefinic and aromatic π receptors. Angew. Chem., Int. Ed..

[37] Gale PA, Howe ENW, Wu X (2016). Anion receptor chemistry. Chem..

[38] García-Garrido SE, Caltagirone C, Light ME, Gale PA (2007). Acridinone-based anion receptors and sensors. Chem. Commun..

[39] Lorenzo A, Aller E, Molina P (2009). Iminophosphorane-based synthesis of multinuclear ferrocenyl urea, thiourea and guanidine derivatives and exploration of their anion sensing properties. Tetrahedron.

[40] Choi JK (2006). A PCT-based, pyrene-armed calix[4]crown fluoroionophore. J. Org. Chem..

[41] Hironori I (2015). Novel 1,8-naphthalimide derivative with an open space for an anion: Unique fluorescence behaviour depending on the anion’s electrophilic. Chem. Commun..

[42] Zhang DW (2013). Acridine-based macrocyclic fluorescent sensors: Self-assembly behavior characterized by crystal structures and a tunable bathochromic-shift in emission induced by H_2_PO_4_^−^ via adjusting the ring size and rigidity. Org. Biomol. Chem..

[43] Bake MV (2004). Azolium-linked cyclophanes: A comprehensive examination of conformations by ^1^H NMR spectroscopy and structural studies. J. Org. Chem..

[44] Kim SK (2003). New fluorescent photoinduced electron transfer chemosensor for the recognition of H_2_PO_4_^−^. Org. Lett..

[45] Hahn FE, Jahnke MC, Pape T (2007). Synthesis of pincer-type bis(benzimidazolin-2-ylidene) palladium complexes and their application in C-C coupling reactions. Organometallics.

[46] Arnold PL, Casely IJ (2009). F-block N-heterocyclic carbene complexes. Chem. Rev..

[47] Hahn FE, Jahnke MC (2008). Heterocyclic carbenes: Synthesis and coordination chemistry. Angew. Chem., Int. Ed..

[48] Liu XL, Chen WZ (2013). Pyridazine-based N-heterocyclic carbene complexes and Ruthenium-catalyzed oxidation reaction of alkenes. Organometallics.

[49] Li Q, Li X, Yang J, Song HB, Tang LF (2013). Synthesis and structural characterization of N-heterocyclic carbene silver complexes derived from N-ferrocenylmethyl-N′-(pyridylmethyl)imidazolium iodides. Polyhedron.

[50] Chen JH, Zhang XQ, Feng Q, Luo MM (2006). Novel hexadentate imidazolium salts in the rhodium-catalyzed addition of arylboronic acids to aldehydes. J. Organomet. Chem..

[51] Sun ZX, Cheng Y (2012). N-Heterocyclic carbene-catalyzed cascade annulation reaction of o-vinylarylaldehydes with nitrosoarenes: One-step assembly of functionalized 2,3-benzoxazin-4-ones. Org. Biomol. Chem..

[52] Liu B, Xia Q, Chen WZ (2009). Direct synthesis of iron, cobalt, nickel, and copper complexes of N-heterocyclic carbenes by using commercially available metal powders. Angew. Chem., Int. Ed..

[53] Zhang F (2013). Synthesis of a novel fluorescent anthryl calix[4]arene as picric acid sensor. Tetrahedron.

[54] Papadopoulou A, Green RJ, Frazier RA (2005). Interaction of flavonoids with bovine serum albumin: A fluorescence quenching study. J. Agric. Food Chem..

[55] Caballero A (2005). Highly selective chromogenic and redox or fluorescent sensors of Hg^2+^ in aqueous environment based on 1,4-disubstituted azines. J. Am. Chem. Soc..

[56] Liu K (2014). Turn on ESPT: Novel salicylaldehyde based sensor for biological important fluoride sensing. J. Photochem. Photobiol. B.

[57] Wang JS, Bodige SG, Watson WH, Gutsche CD (2000). Complexation of fullerenes with 5,5′-biscalix[5]arene. J. Org. Chem..

[58] Polster, J. & Lachmann, H. Spectrometric titrations: VCH, Weinheim. 256–259 (1989).

[59] Cao MJ (2015). A dansyl-based fluorescent probe for selectively detecting Cu^2+^ and imaging in living cells. RSC Adv..

[60] Liu QX (2011). Two N-heterocyclic carbene silver(I) cyclophanes: Synthesis, structural studies, and recognition for p-phenylenediamine. Organometallics.

[61] Shang XF (2013). Colorimeric and fluorescence ON-OFF probe for acetate anion based on thiourea derivative: Theory and experiment. Spectrochimica Acta A.

[62] SMART 5.0 and SAINT 4.0 for Windows NT, Area Detector Control and Integration Software, Bruker Analytical X-Ray Systems, Inc., Madison, WI, USA (1998).

[63] Sheldrick, G. M. SADABS, Program for Empirical Absorption Correction of Area Detector Data, Univ. of Göttingen, Germany (1996).

[64] Sheldrick, G. M. SHELXTL 5.10 for Windows NT, Structure Determination Software, Brucker Analytical X-Ray Systems, Inc., Madison, WI, USA (1997).

