# Supplementary material for: Two macrocycle-based sensors for anions sensing
Source: Sci Rep. 2019 Jan 24;9:502. doi: 10.1038/s41598-018-36916-w (PMC6345996; doi:10.1038/s41598-018-36916-w)
Supplement: Supplementary file 1 — Supplementary Information [file 41598_2018_36916_MOESM1_ESM.pdf]

SUPPLEMENTARY INFORMATION

Two macrocycle-based sensors for anions sensing

Yingjie Liu,[a] Zhixiang Zhao[b], Ran Huo[b], Qingxiang Liu[b*]

[a]*Tianjin Key Laboratory of Process Measurement and Control, Institute of Robotics and Autonomous Systems, Tianjin University, Tianjin 300072, China*

[b]*Key Laboratory of Inorganic-Organic Hybrid Functional Materials Chemistry (Tianjin Normal University), Ministry of Education; Tianjin Key Laboratory of Structure and Performance for Functional Molecules, College of Chemistry, Tianjin Normal University, Tianjin 300387, China*

** Corresponding author, E-mail: tjnulqx@163.com; Postal address: Binshuixi Road No.393, Xiqing District, Tianjin City, 300387, China*

Table of Contents

1. CCDC number for **2** and **4**

2. Summary of crystallographic data for **2** and **4** (Table S1)

3. The UV/Vis and fluorescence studies of **2** and **4** (Figure S1-Figure S13)

4. HRMS spectra for **2**·OAc^- and **4**·NO_3^- (Figure S14 and Figure S 15)

5. The infrared spectra of **2**, **2**·OAc^- and **4**, **4**·NO_3^- (Figure S16 and Figure S17)

6. The ^{1}H NMR and ^{13}C NMR spectra of intermediates and compounds **2** and **4** (Figure S18-Figure S26)

1. CCDC Number for 2 and 4.

CCDC 1553466 and 1557478 contain the supplementary crystallographic data for macrometallocycle **2** and **4**. These data can be obtained free of charge via http//www.ccdc.cam.ac.uk/conts/retrieving.html, or from the Cambridge Crystallographic Data Centre, 12 Union Road, Cambridge, CB2 1EZ, UK; fax: (+44) 1223-336-033; or e-mail: deposit@ccdc.cam.ac.uk.

2. Summary of Crystallographic Data for 2 and 4.

Table S1. Summary of crystallographic data for **2** and **4**

	**2**	**4**
Chemical formula	$C_{44}H_{40}F_{12}N_4O_4P_2$	$C_{46}H_{44}F_{12}N_4O_4P_2$
Fw	978.74	1006.79
Cryst syst	Triclinic	Orthorhombic
Space group	$P\bar{1}$	*Pbcn*
a/Å	9.881(3)	15.894(3)
b/Å	15.483(6)	18.582(3)
c/Å	16.931(7)	36.764(7)
α/deg	99.6(3)	90
β/deg	94.5(3)	90
γ/deg	97.1(3)	90
V/Å^3	2520.5(1)	10858.5(3)
Z	2	8
D_{calcd}, Mg/m^3	1.290	1.232
Abs coeff, mm^{-1}	1.557	1.459
F(000)	1004	4144
Cryst size, mm	0.25 × 0.20 × 0.15	0.25 × 0.24 × 0.23
θ_{min}, θ_{max}, deg	3.58, 67.07	3.659, 74.508
T /K	173	293(2)
No. of data collected	17062	29705
No. of unique data	8974	10849
No. of refined params	605	630
Goodness-of-fit on F^{2} [a]	1.083	1.081
Final R indices[b] [$I > 2\sigma(I)$]		
R1	0.0658	0.0828
wR2	0.1886	0.2467
R indices (all data)		
R1	0.0772	0.0954
wR2	0.2015	0.2588

^{a}GOF = $[\Sigma\omega(F_o^2 - F_c^2)^2 /(n - p)]^{1/2}$, where n is the number of reflection and p is the number of parameters refined. $^bR_1 = \Sigma(||F_o| - |F_c||)/\Sigma|F_o|$; $wR_2 = [\Sigma[w(F_o^2 - F_c^2)^2]/ \Sigma w(F_o^2)^2]^{1/2}$.

3. The Ultraviolet and Fluorescence Studies of Compounds 2 and 4.

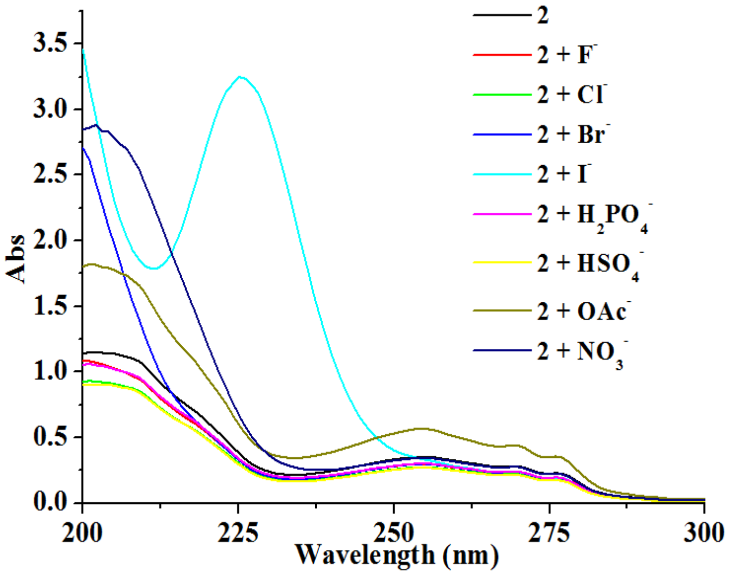

Figure S1. Ultroviolet absorption spectra of **2** (1×10^{-5} M) and upon the addition of salts (20.0 equiv.) of F^-, Cl^-, Br^-, I^-, $H_2PO_4^-$, HSO_4^-, OAc^- and NO_3^-, and their cations being tetrabutyl ammonium (TBA^+) in H_2O/CH_3CN (v:v = 1:1) at 25 °C.

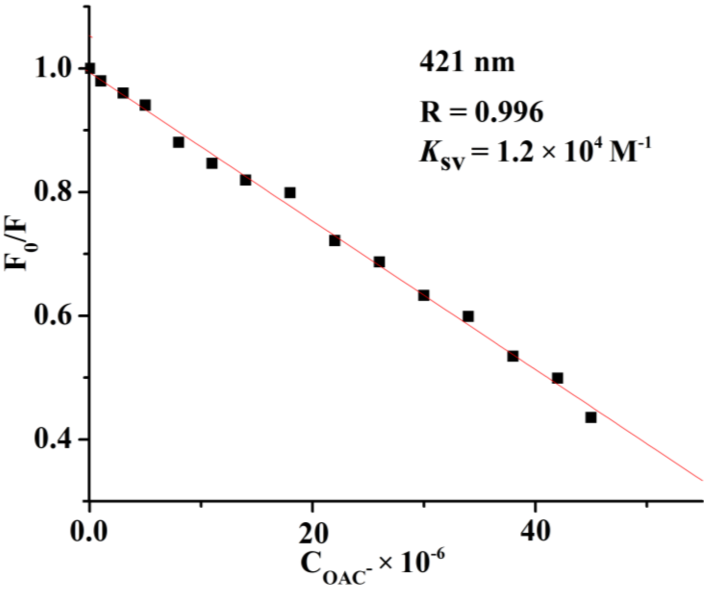

Figure S2. Stern-Volmer plot describing **2** increasing caused by OAc^- association in H_2O/CH_3CN solutions at 421 nm. The K_{SV} is 1.2×10^4 M^{-1}, and the linear range is

from 0-44 $\times 10^{-6}$ M.

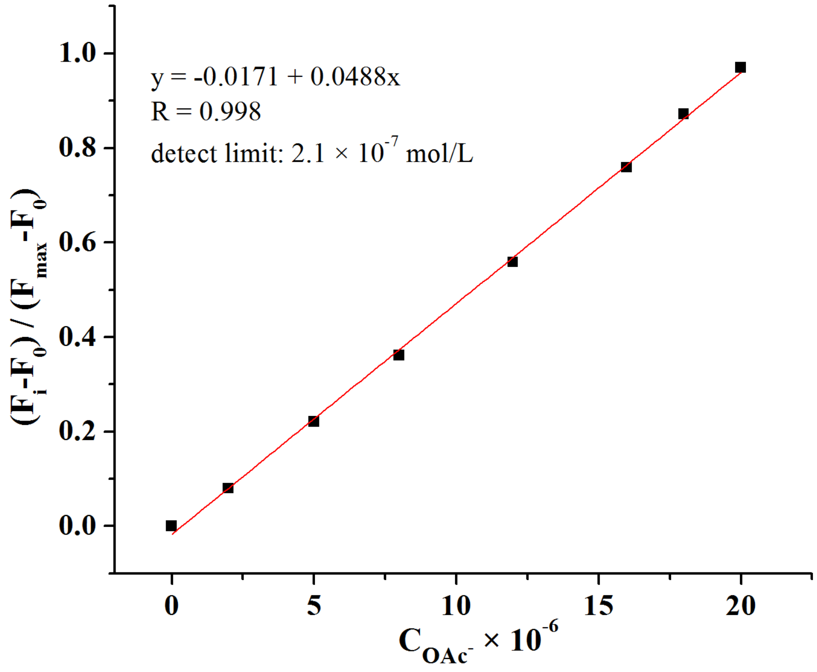

Figure S3. Emission (at 421 nm) of **2** at different concentrations of OAc^- (0, 2, 5, 8, 12, 16, 18, 20 μM) added, normalized between the minimum emission (0.0 μM OAc^-) and the emission at 20 μM OAc^-. The detection limit was determined to be 2.1 $\times 10^{-7}$ M.

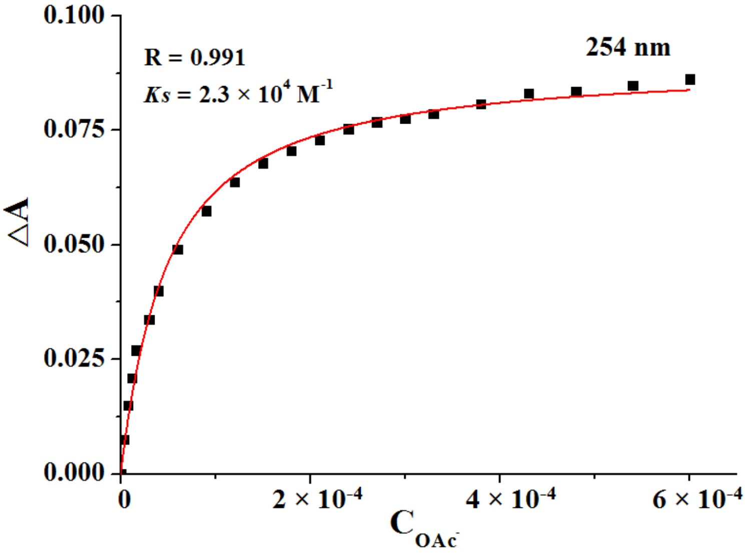

Figure S4. Non-linear relationship between ΔA versus C_{OAc^-} at 254 nm for **2**.

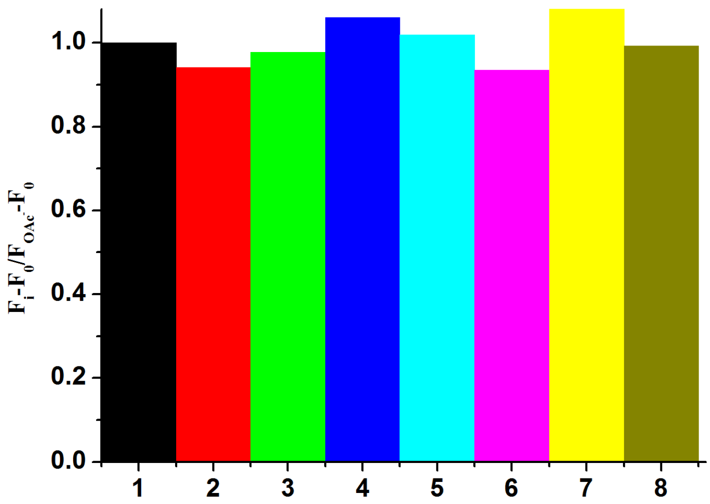

Figure S5. Change ratio ((F_i - F_0)/(F_{OAc-} - F_0) of fluorescence intensity of **2** upon addition of 20 equiv. of OAc^- in the presence of 20 equiv. of background anions. 1: OAc^-; 2: OAc^- + F^-; 3: OAc^-+ Cl^-; 4: OAc^- + Br^-; 5: OAc^- + I^-; 6: OAc^- + $H_2PO_4^-$; 7: OAc^- + HSO_4^-; 8: OAc^- + NO_3^- in H_2O/CH_3CN (v:v = 1:1) at 25 ˚C.

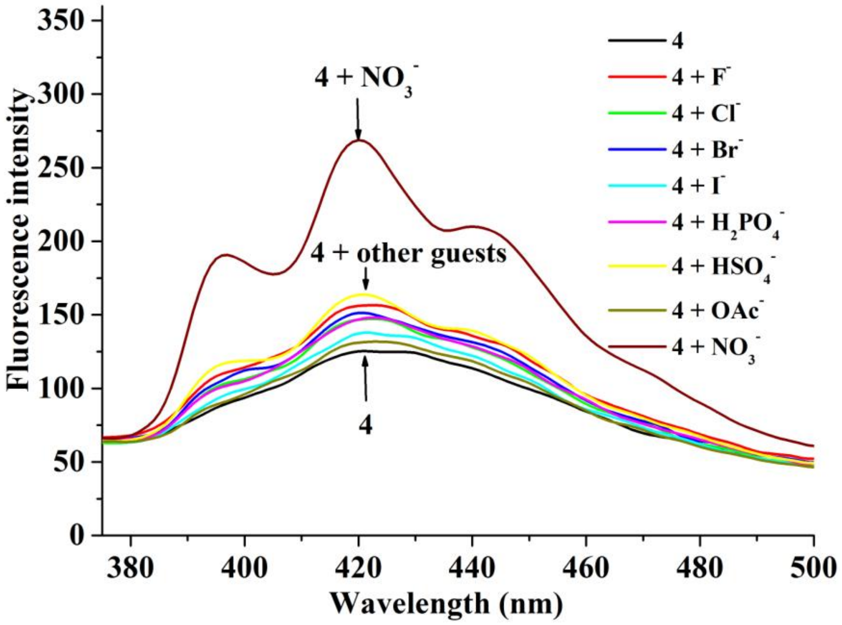

Figure S6. Fluorescence spectra of **4** (1 × 10^{-6} mol/L) and upon the addition of salts (20 equiv.) of F^-, Cl^-, Br^-, I^-, $H_2PO_4^-$, HSO_4^-, OAc^- and NO_3^-, and their cations being tetrabutyl ammonium (TBA^+) in CH_3CN/H_2O at 25 ˚C.

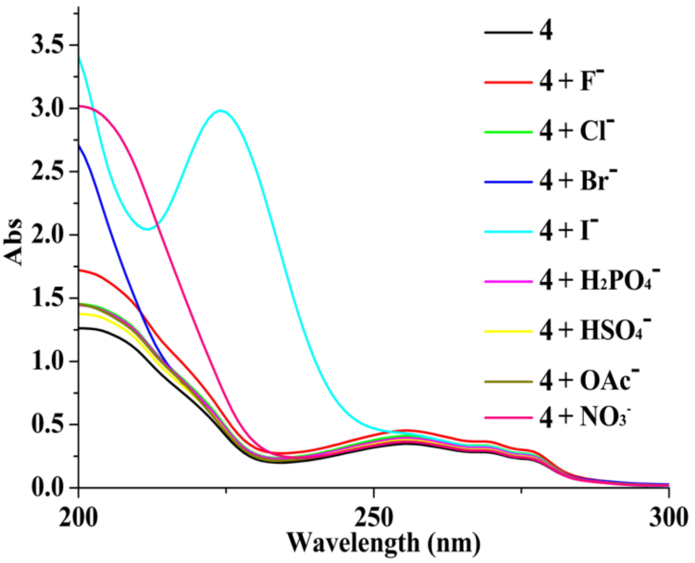

Figure S7. UV-vis absorption spectra of **4** (1×10^{-5} mol/L) and upon the addition of salts (20.0 equiv.) of F^-, Cl^-, Br^-, I^-, $H_2PO_4^-$, HSO_4^-, OAc^- and NO_3^-, and their cations being tetrabutyl ammonium (TBA^+) in H_2O/CH_3CN at 25 ˚C.

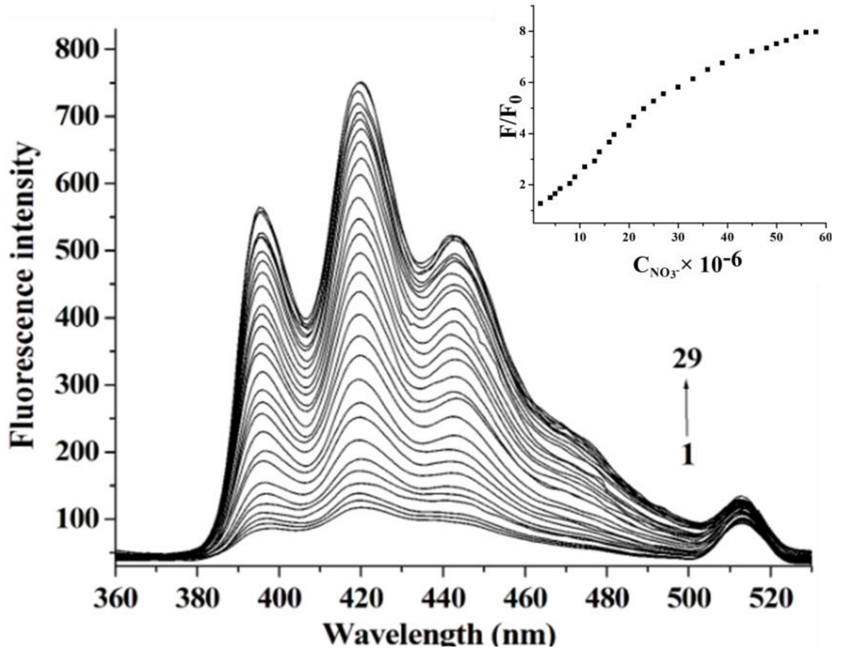

Figure S8. Fluorescence titration spectra of **4** (1.0×10^{-6} mol/L) in the presence of different concentrations of NO_3^- in H_2O/CH_3CN at 25 ˚C. $C_{NO_3^-}$ for curves 1-29 (from bottom to top) are 0, 2, 4, 5, 6, 8, 9, 11, 13, 14, 16, 17, 20, 21, 23, 25, 27, 30, 33, 36, 39, 42, 45, 48, 50, 52, 54, 56, 58 $\times 10^{-6}$ mol/L (λ_{ex} = 254 nm). Inset: the fluorescence at 421 nm of **4** as a function of NO_3^- concentration.

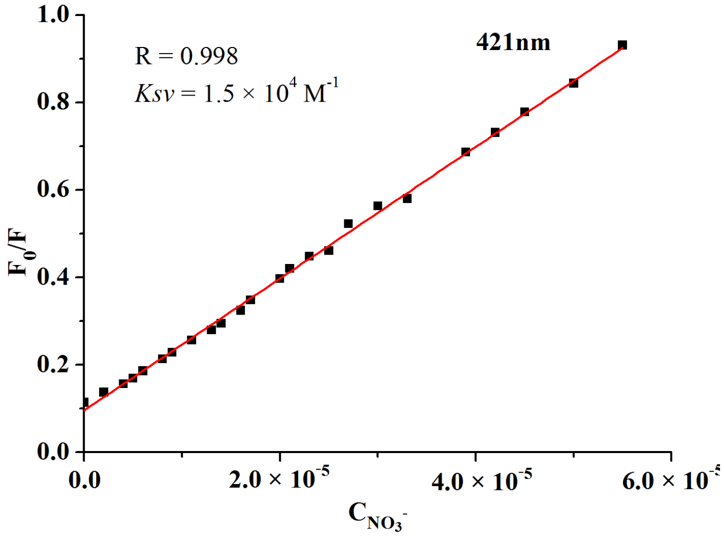

Figure S9. Stern-Volmer plot of host **4** rised by NO_3^- in H_2O/CH_3CN solutions at 421 nm. The K_{SV} is 1.5×10^4 M^{-1}, and the linear range is from 0-60 $\times 10^{-6}$ mol/L.

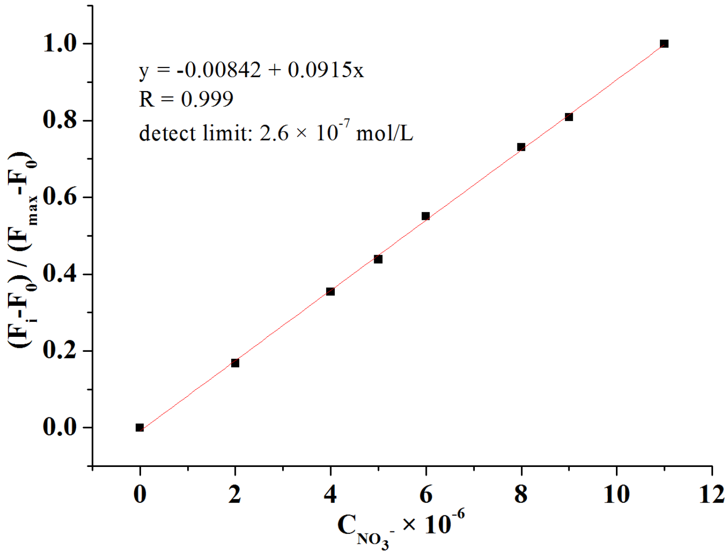

Figure S10. Emission (at 421 nm) of **4** at different concentrations of NO_3^- (0, 2, 4, 5, 6, 8, 9, 11 μM) added, normalized between the minimum emission (0.0 μM NO_3^-) and the emission at 11 μM NO_3^-. The detection limit was determined to be 2.6 $\times 10^{-7}$ M.

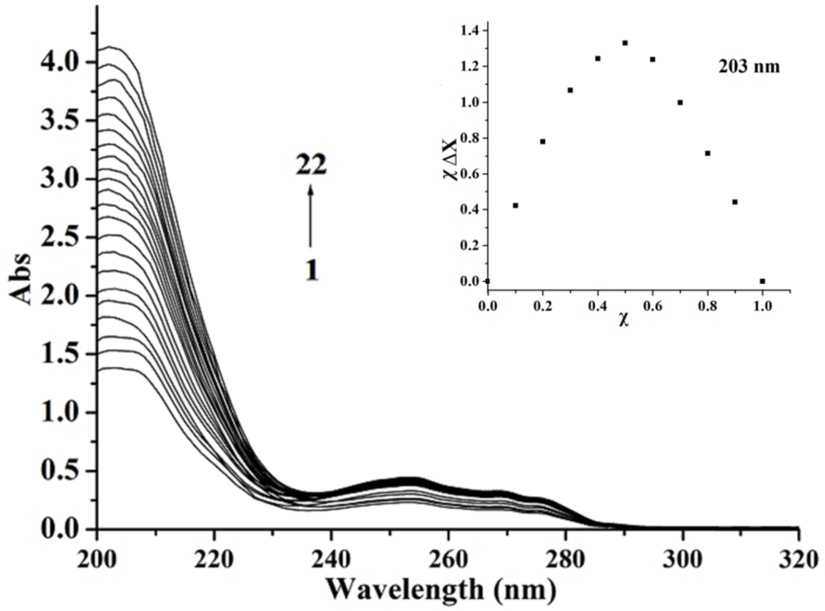

Figure S11. UV/vis absorption spectra of **4** in CH_3CN/H_2O at an invariant total concentration of 1×10^{-5} mol/L at 25 °C. The concentrations of NO_3^- for curves 1-22 (from top to bottom) are: 0, 0.4, 0.8, 1.2, 1.6, 3, 4, 6, 9, 12, 15, 18, 21, 24, 27, 30, 33, 38, 43, 48, 54, 60 $\times 10^{-5}$ mol/L. Inset: the Job's plot for a **4**·NO_3^- complex at 203 nm. χ is molar fractions of **4**, and $\chi\Delta A$ is the products between molar fractions and the discrepancy of the absorption bands.

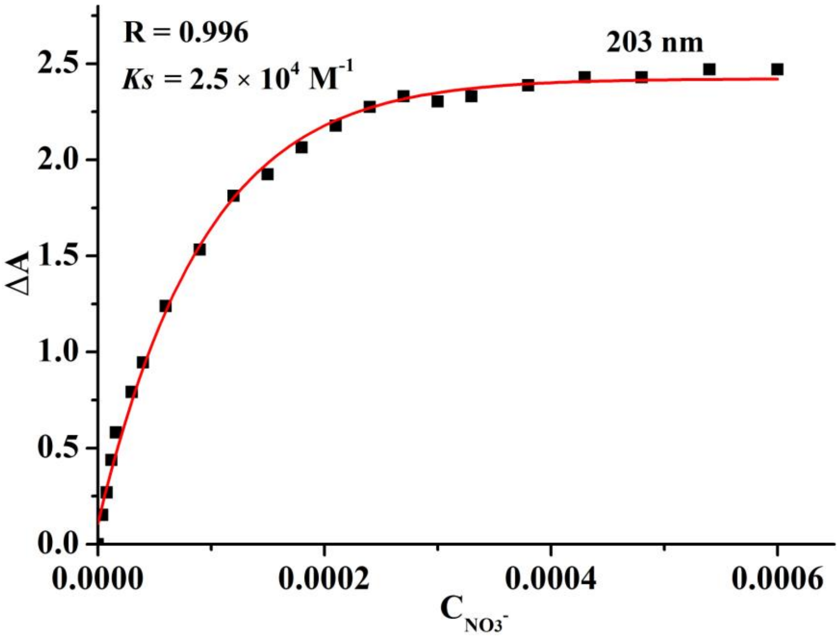

Figure S12. Non-linear relationship between ΔA versus $C_{NO_3^-}$ at 203 nm for **4**.

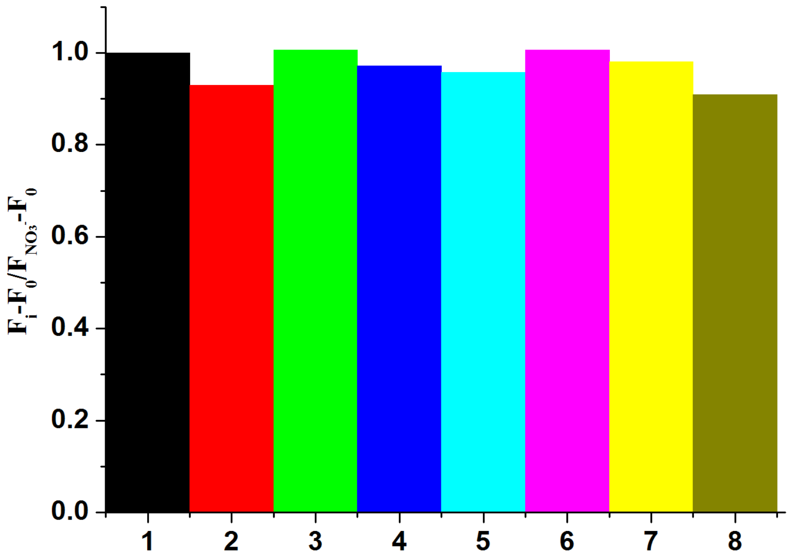

Figure S13. Change ratio (F_i - F_0)/($F_{NO_3^-}$ - F_0) of fluorescence intensity of **4** upon addition of 20 equiv. NO_3^- in the presence of 20 equiv. of background anions. 1: NO_3^-; 2: NO_3^- + F^-; 3: NO_3^- + Cl^-; 4: NO_3^- + Br^-; 5: NO_3^- + I^-; 6: NO_3^- + $H_2PO_4^-$; 7: NO_3^- + HSO_4^-; 8: NO_3^- + OAc^- in H_2O/CH_3CN at 25 ˚C.

4. HRMS Spectra for 2·OAc$^-$ and 4·NO$_3^-$.

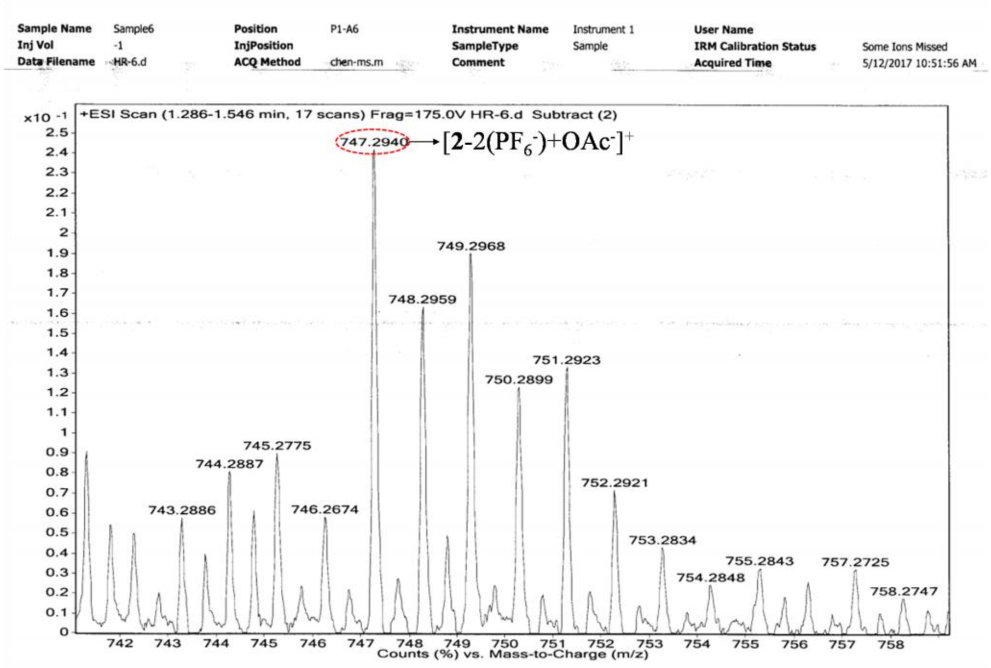

Figure S14. HRMS spectra for **2**·OAc$^-$. MS (EI): m/z [**2**-2(PF_6^-)+OAc^-]$^+$ = 747.2940.

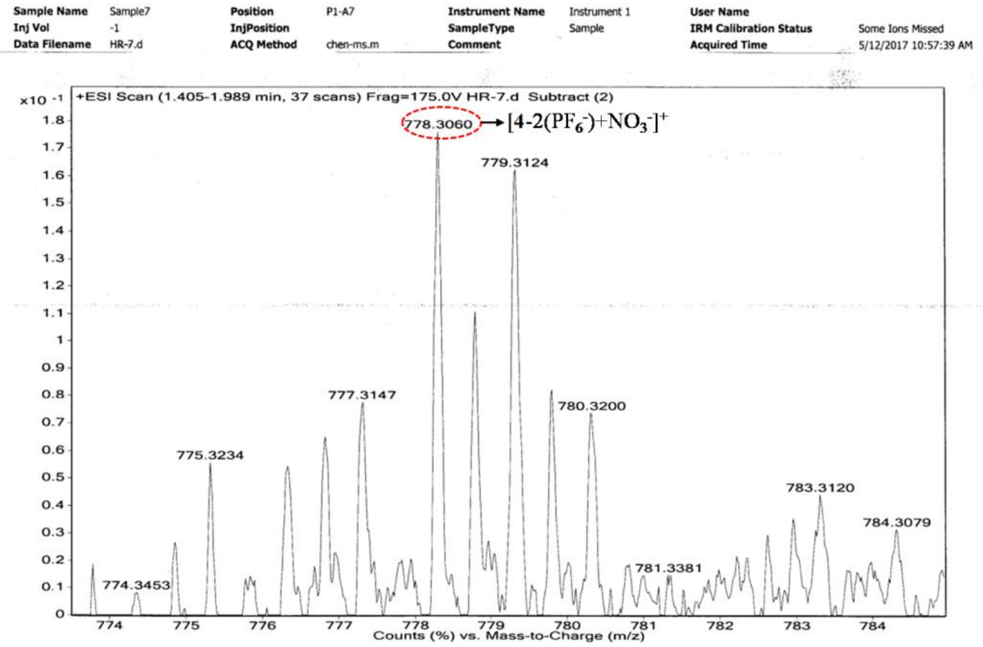

Figure S15. HRMS spectra for **4**·NO_3^-. MS (EI): m/z [**4**-2(PF_6^-)+NO_3^-]$^+$ = 778.3060.

5. The Infrared Spectra of 2, 2·OAc$^-$ and 4, 4·NO_3^-.

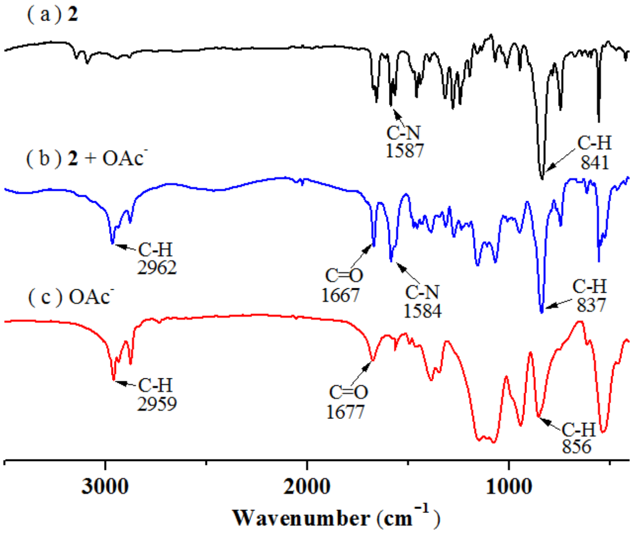

Figure S16. Infrared spectra of (a) **2**; (b) **2**·OAc$^-$; (c) OAc$^-$.

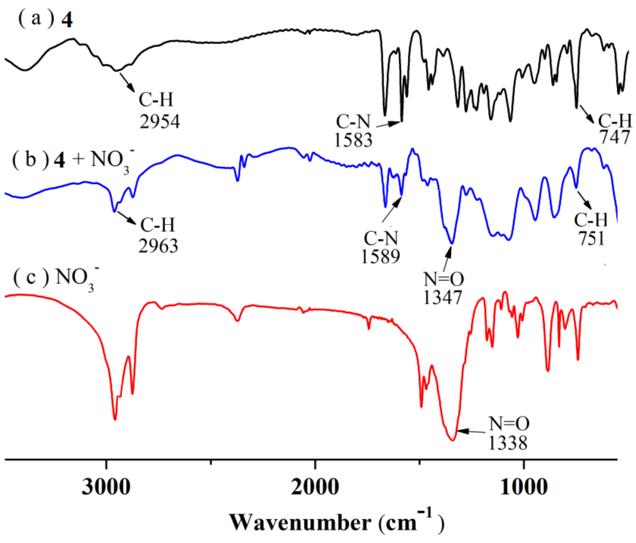

Figure S17. Infrared spectra of (a) **4**; (b) **4** ·NO_3^-; (c) NO_3^-.

6. The ^{1}H NMR and ^{13}C NMR Spectra of Intermediates and Compounds 2 and 4.

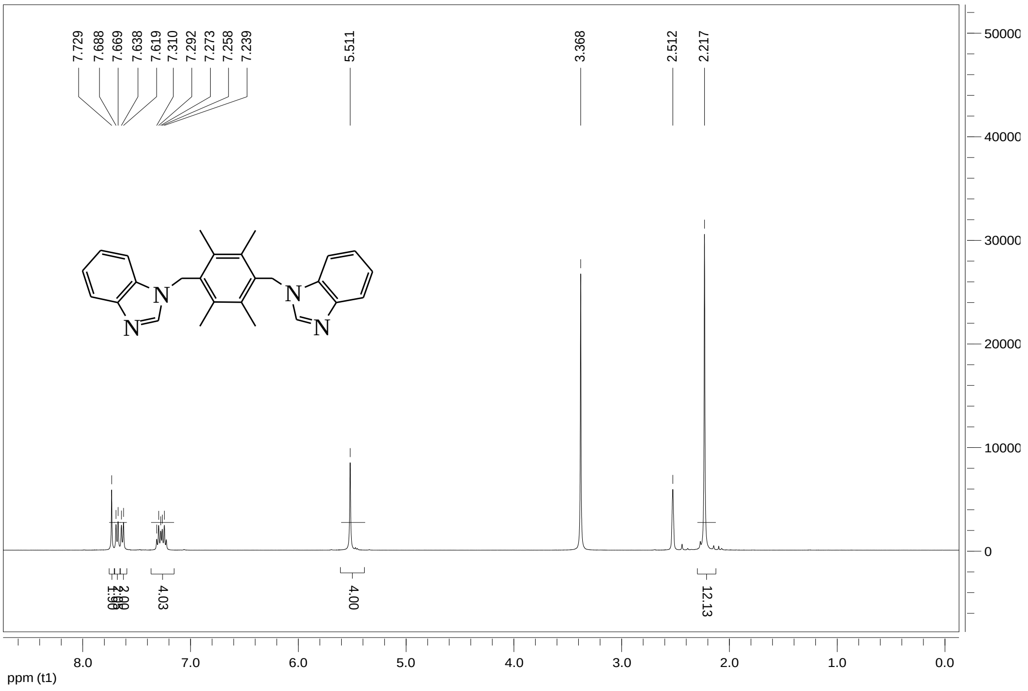

Figure S18. The ^{1}H NMR (400 MHz, DMSO-d_6) spectra of 1,4-di(benzimidazole-methyl)-durene.

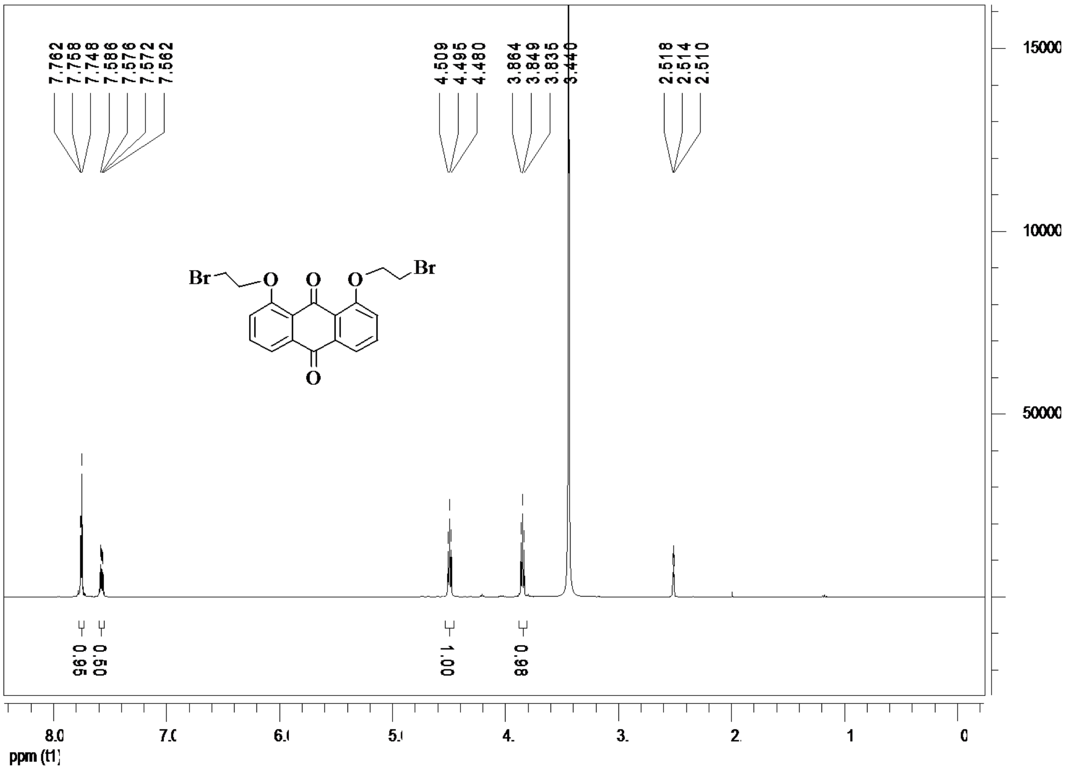

Figure S19. The ^{1}H NMR (400 MHz, DMSO-d_6) spectrum of 1,8-bis(2’-bromoethoxy)-9,10-anthraquinone.

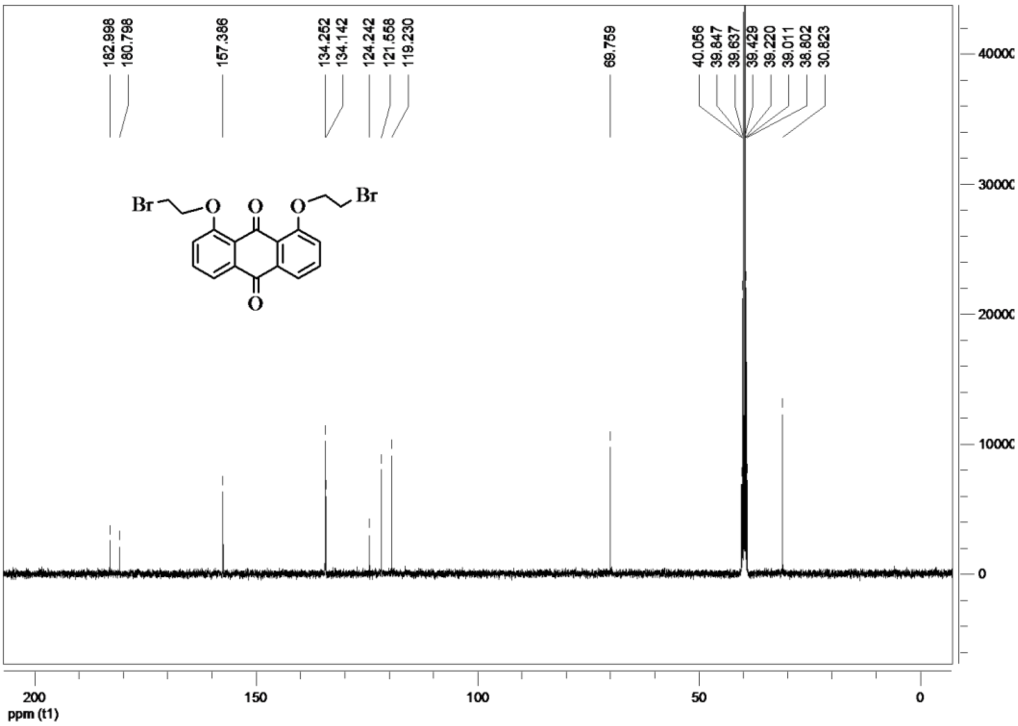

Figure S20. The ^{13}C NMR (100 MHz, DMSO-d_6) spectrum of 1,8-bis(2’-bromoethoxy)-9,10-anthraquinone.

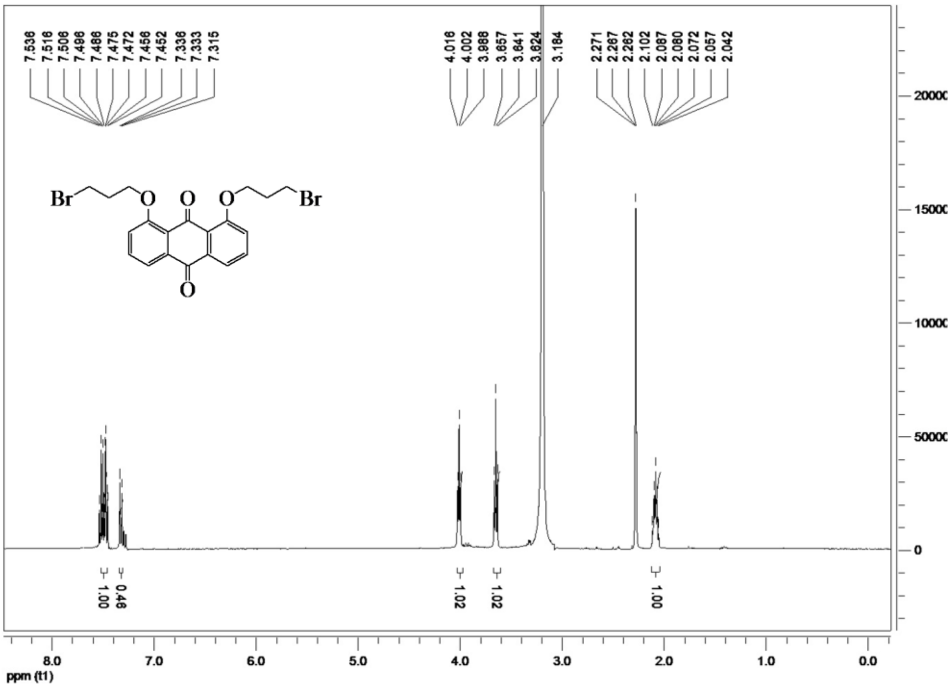

Figure S21. The ^{1}H NMR (400 MHz, DMSO-d_6) spectra of 1,8-bis(3'-bromoethoxy)-9,10-anthraquinone.

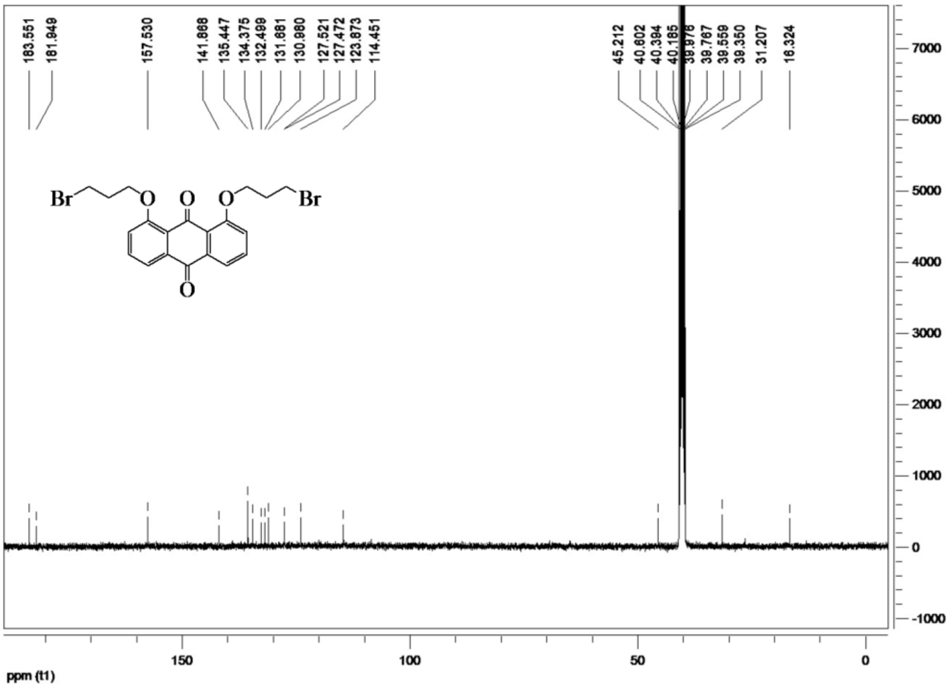

Figure S22. The ^{13}C NMR (100 MHz, DMSO-d_6) spectrum of 1,8-bis(2-bromoethoxy)-9,10-anthraquinone.

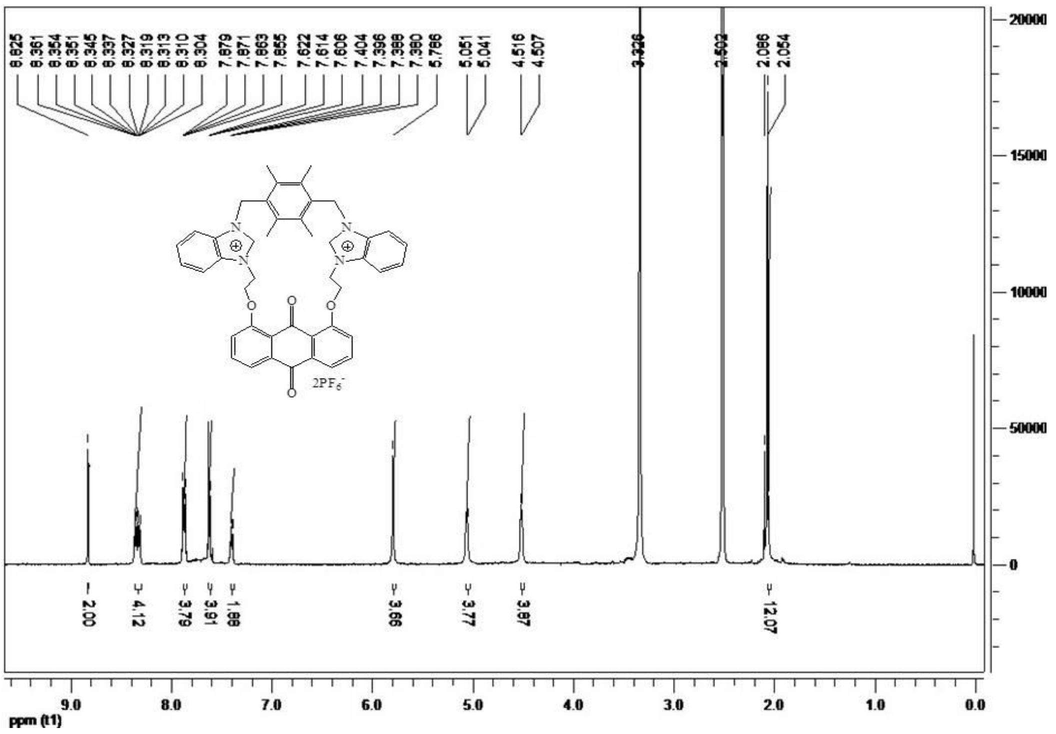

Figure S23. The ^{1}H NMR (400 MHz, DMSO-d_6) spectra of **2**.

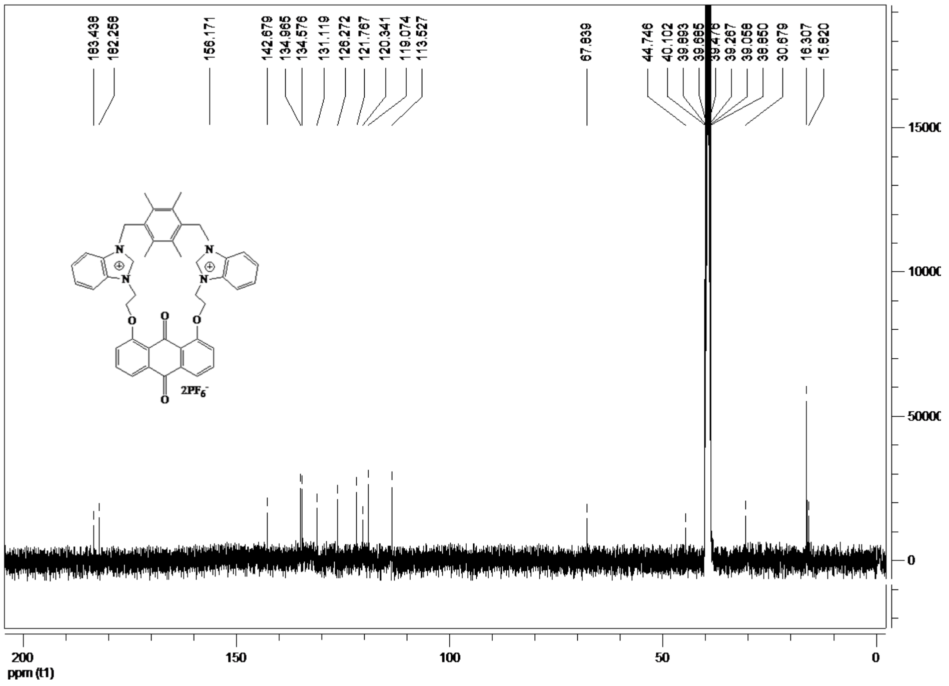

Figure S24. The ^{13}C NMR (100 MHz, DMSO-d_6) spectra of **2**.

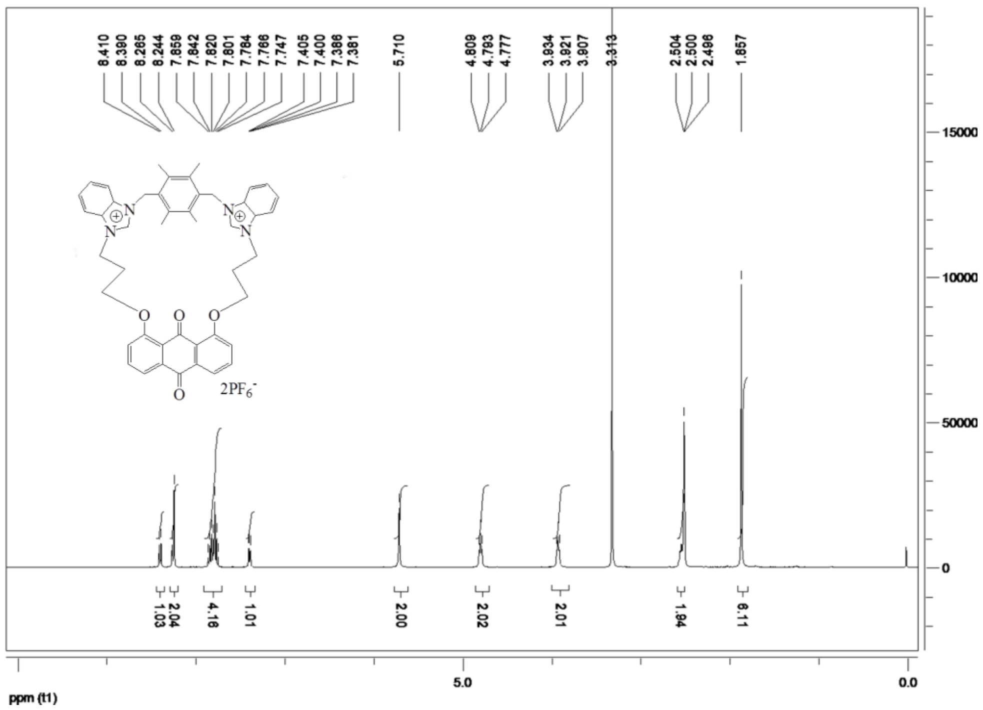

Figure S25. The ^{1}H NMR (400 MHz, DMSO-d_6) spectra of **4**.

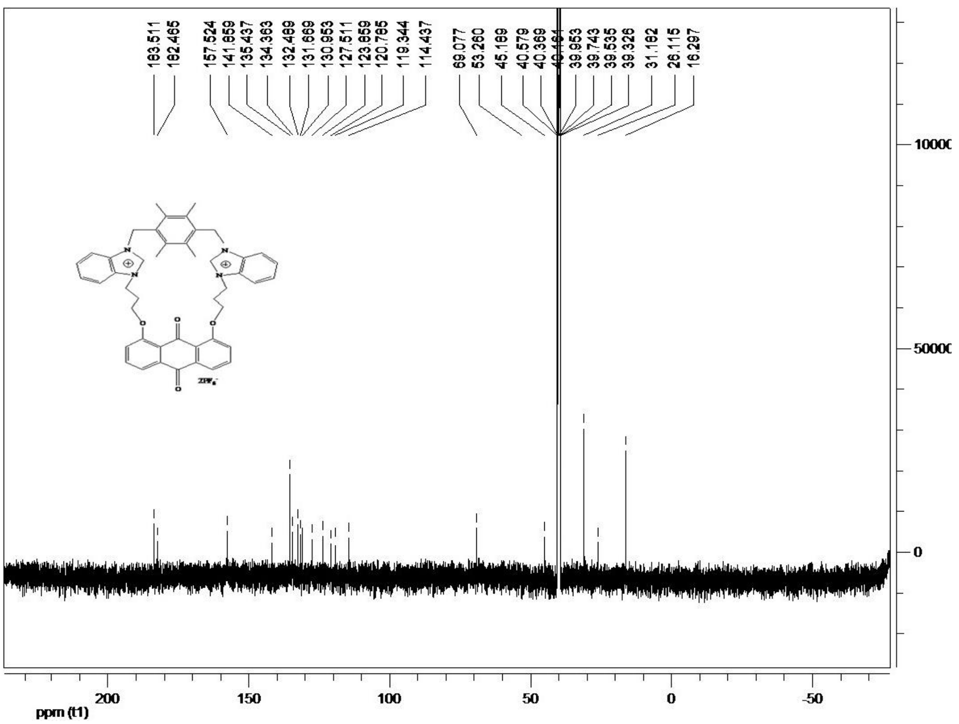

Figure S26. The ^{13}C NMR (100 MHz, DMSO-d_6) spectra of **4**.
